# The RAD52-like protein ODB1 is required for the efficient excision of two mitochondrial introns spliced via first-step hydrolysis

**DOI:** 10.1093/nar/gkv540

**Published:** 2015-06-05

**Authors:** José M. Gualberto, Monique Le Ret, Barbara Beator, Kristina Kühn

**Affiliations:** 1 Institut de Biologie Moléculaire des Plantes-CNRS-UPR2357, Université de Strasbourg, Strasbourg, France; 2 Molekulare Zellbiologie der Pflanzen, Institut für Biologie, Humboldt-Universität zu Berlin, 10115 Berlin, Germany

## Abstract

Transcript splicing in plant mitochondria involves numerous nucleus-encoded factors, most of which are of eukaryotic origin. Some of these belong to protein families initially characterised to perform unrelated functions. The RAD52-like ODB1 protein has been reported to have roles in homologous recombination-dependent DNA repair in the nuclear and mitochondrial compartments in *Arabidopsis thaliana*. We show that it is additionally involved in splicing and facilitates the excision of two *cis*-spliced group II introns, *nad1* intron 2 and *nad2* intron 1, in Arabidopsis mitochondria. *odb1* mutants lacking detectable amounts of ODB1 protein over-accumulated incompletely spliced *nad1* and *nad2* transcripts. The two ODB1-dependent introns were both found to splice via first-step hydrolysis and to be released as linear or circular molecules instead of lariats. Our systematic analysis of the structures of excised introns in Arabidopsis mitochondria revealed several other hydrolytically spliced group II introns in addition to *nad1* intron 2 and *nad2* intron 1, indicating that ODB1 is not a general determinant of the hydrolytic splicing pathway.

## INTRODUCTION

Higher plant organelle genomes contain several group-II introns originating from mobile ribozymes that invaded these genomes during evolution (1–3). Plant organellar introns cannot self-splice; their removal from pre-mRNAs requires the participation of intron maturases and other protein cofactors, most of which are nuclear-encoded (4–6). Group II introns in plant mitochondria are found exclusively in protein-coding genes, with the majority residing in genes for subunits of respiratory chain complex I (1). Plants in which mitochondrial mRNA splicing is disrupted due to loss of a crucial splice factor are usually defective in electron transport chain biogenesis and display severe respiratory disturbances (4).

Components of the plant mitochondrial splicing machinery have mostly been identified by homology to intron-encoded maturases or previously characterised chloroplast splice factors and through analyses of loss-of-function mutants in *Arabidopsis thaliana*. They can be assigned to two categories: (i) Intron-encoded RNA maturases and related maturase enzymes encoded by nuclear genes, and (ii) splicing factors proposed to have originated from host proteins and to have been recruited to additionally function in mitochondrial transcript processing (1). For three out of four nuclear-encoded maturases in Arabidopsis, nMAT1, nMAT2 and nMAT4, participation in mitochondrial intron splicing has been established (7–9). In contrast, a role for the single mitochondrial intron-encoded maturase, MatR, in the splicing of its cognate intron or other mitochondrial introns remains to be demonstrated. nMAT1, nMAT2 and nMAT4 each are required for the splicing of three different and apparently unrelated introns. While the specific biochemical functions of nuclear-encoded maturases in mitochondrial group-II intron splicing are yet to be defined, nMAT1 has been suggested to be required for the 5′ exon release from the introns whose splicing it mediates (4). Other splice factors identified in higher plant mitochondria include DEAD and DExH-box RNA helicases (10,11), which function as RNA chaperones needed to resolve stable inactive secondary structures and promote correct intron folding (3). Arabidopsis plants lacking these helicases are defective in the splicing of multiple mitochondrial introns. The majority of known mitochondrial splice factors are RNA-binding proteins that potentially stabilise the intron RNA structure (1). Among these, pentatricopeptide repeat (PPR) proteins, which bind to specific RNA sequences (12–14), make up the largest group. The PPR proteins OTP43, BIR6, ABO5, OTP439 and TANG2 each are required for the splicing of a specific mitochondrial intron (15–18). The PPR protein MTSF1, which functions primarily in *nad4* mRNA 3′-end maturation and whose absence strongly destabilises the *nad4* mRNA, additionally facilitates the splicing of the first *nad2* intron in Arabidopsis mitochondria (19). Other RNA-binding proteins with a role in mitochondrial transcript splicing are the PORR (plant organellar RNA recognition) domain protein WTF9, which is specifically required for intron excision from the *rpl2* and *ccmFc* pre-mRNAs (20), and the CRM (chloroplast RNA splicing and ribosome maturation) domain protein mCSF1 involved in the splicing of multiple mitochondrial introns (21). Perhaps the most unusual mitochondrial splice factor identified to date is RUG3 (RCC1/UVR8/guanine exchange factor-like 3) (22). This protein is related to plant and animal chromatin-binding factors functioning in the nucleus and facilitates splicing of the third *nad2* intron. Removal of this intron additionally depends on the mitochondrial transcription termination factor (mTERF) family protein mTERF15 (23). Excepting RUG3, all identified mitochondrial splice factors have known homologues functioning in chloroplast group II intron splicing (5 and references therein; 24–26).

Group II introns have a distinctive secondary structure with six domains (dI-dVI) extending from a central hub; tertiary interactions between these domains mediate productive intron folding (reviewed in 3,27,28). dV and dVI are particularly important for the biochemistry of splicing, the conserved dV forming part of the catalytic core of the ribozyme and dVI containing an unpaired adenosine which functions as attacking nucleophile during the first step of splicing. Classical group II introns splice via two sequential transesterifications, the first of which covalently joins the dVI unpaired adenosine, named ‘bulging A’, to the intron 5′ end and results in the formation of a lariat in which the bulging A is the branch-point nucleotide (3). The second transesterification joins the two exons, thus releasing the intron lariat. An alternative group II intron splicing mechanism exists where the first step of splicing occurs by hydrolysis. It was initially observed only *in vitro* (29,30) but subsequently shown to occur *in vivo* in yeast for introns with branch-point mutations (31). In seed plants, where several organellar introns deviate considerably from the group II consensus and lack a conventional branch-point nucleotide, splicing via first-step hydrolysis was first shown for the chloroplast *trnV* intron (32) and later demonstrated for three different mitochondrial introns (33,34). Hydrolytically spliced introns were found to be released as linear or circular molecules. Intron excision from plant mitochondrial transcripts involves both *cis*- and *trans*-splicing (35). Extensive mis-splicing has been reported for the mitochondrial *nad5* transcript, which is transcribed from three different genomic regions, in certain plant species (36).

This study reports on the involvement of the Arabidopsis RAD52-like protein ODB1 in the *cis*-splicing of two specific mitochondrial introns, *nad1* intron 2 and *nad2* intron 1, both of which are released via a hydrolytic pathway. ODB1 was found earlier to have a role in homologous recombination (HR)-dependent DNA repair in the nuclear and mitochondrial compartments; it co-localised with mitochondrial high-molecular weight complexes in a DNA- and RNA dependent manner (37,38). Our systematic analysis of intron splicing pathways in Arabidopsis mitochondria revealed several other hydrolytically spliced introns in addition to *nad1* intron 2 and *nad2* intron 1, indicating that ODB1 is not a general determinant of the hydrolytic splicing pathway.

## MATERIALS AND METHODS

### Plant material

*Arabidopsis thaliana* wild-type plants (ecotype Columbia Col-0), T-DNA insertion lines *odb1–1* (Sail_25_H08) and *odb1–2* (SALK_089362) (37) and complemented lines were grown on soil at 23°C in a 16-h photoperiod at a light intensity of 100 mmol quanta m^−2^ s^−1^. For nucleic acid extractions from seedlings, surface-sterilised seeds were sown on agar plates containing MS255 (Duchefa) and supplemented with 1% w/v sucrose, stratified at 4°C for 3 days in the dark and then exposed to a light intensity of 100 μmol quanta m^−2^ s^−1^ in a 16-h photoperiod at 23°C.

### DNA and RNA isolation and reverse transcriptase-polymerase chain reaction (RT-PCR)

Genomic DNA was isolated from seedlings or flowers as previously described (39). RNA was extracted from seedlings grown on agar for 7 d or from flowers of soil-grown plants using TRI Reagent (Molecular Research Centre, Inc.). For all subsequent procedures except RNA gel blot hybridisations, RNA samples were depleted in contaminating genomic DNA by treatment with RQ1 RNase-free DNase (Promega) and confirmed by PCR to be free of detectable amounts of DNA. To produce cDNA for quantitative RT-PCR experiments and Northern blot probe synthesis, 3 μg of RNA were reverse-transcribed with Superscript III Reverse Transcriptase (Life Technologies) according to the manufacturer's protocol using random hexamers.

### *odb1* mutant complementation

A genomic DNA fragment comprising the *ODB1* promoter, coding region and untranslated regions was amplified using Phusion DNA polymerase (Finnzymes) and primers 5′- GGGGACAAGTTTGTACAAAAAAGCAGGCTTGTTATTAAGTAACGTCCTGCCA-3′ and 5′- GGGGACCACTTTGTACAAGAAAGCTGGGTTGTTTGTTAAGTCGAATAACCACC-3′ and transferred into binary vectors pKWG (complemented line C1) or pGWB40 (complemented line C2) using gateway cloning procedures (Life Technologies). The resulting plasmids were used to transform *odb1–1* plants via Agrobacterium-mediated floral dip transformation. Resulting seeds were sown on soil and transformants selected using kanamycin (pKWG) or hygromycin (pGWB40). *odb1–1* plants carrying the *ODB1* transgene were identified by PCR using primers amplifying the vector-insert junctions.

### RNA gel blot analysis

Total RNA (3 μg) extracted from Arabidopsis seedlings was resolved on 1.2% w/v agarose/formaldehyde gels and transferred onto positively charged nylon membranes (Roche Applied Science), followed by membrane staining with methylene blue (0.04% (w/v) in 0.5M sodium acetate, pH 5.2). DIG-labelled DNA probes were generated using Arabidopsis genomic DNA or cDNA as template, the PCR DIG probe synthesis kit (Roche Applied Science), and primer pairs listed in Supplementary Table S1. Hybridisations were performed in DIG Easy Hyb solution (Roche Applied Science) according to the manufacturer's instructions; chemiluminescent detection of signals was performed using anti-digoxigenin-alkaline phosphatase conjugates and CSPD reagent (Roche Applied Science).

### Real-time quantitative RT-PCR

Quantitative RT-PCR (qRT-PCR) for measuring mitochondrial intron splicing efficiencies was performed using SYBR Green I Master Mix (Roche Applied Science) and primer pairs listed in Supplementary Table S2 as previously described (16) on a LightCycler 480 real-time PCR system (Roche Applied Science). The nuclear *18S* rRNA (At3g41768) and *ACT* genes (At1g49240, At3g18780) were used for data normalisation.

### Mapping of *nad2* transcript extremities by cRT-PCR

RNA (1 μg) was incubated with 2.5 units of tobacco acid pyrophosphatase (TAP, Epicentre) as previously described (40); control reactions were set up without TAP. Transcripts were subsequently extracted with phenol/chloroform/isoamyl alcohol (25:24:1), precipitated from the aqueous phase by adding 3 volumes of ethanol/3 M sodium acetate, pH 5.2 (30:1) and dissolved in water. The RNA was then treated with 40 U of T4 RNA ligase (New England Biolabs) at 37°C for 1 h in the presence of 1 mM ATP and 40 U of RNase inhibitor (Life Technologies) in the appropriate buffer. RNA was extracted from ligation reactions and precipitated as before, dissolved in water and then reverse-transcribed using a *nad2* intron 1-specific primer (5′-GAAAGCCCGGCCAGAAC-3′) and SuperScript III Reverse Transcriptase (Life Technologies). The resulting cDNAs served as template in subsequent PCR reactions set up with primers 5′-GGACTCATGTGCCTTCCAGAAC-3′ and 5′-ATGAACCAATGGATCGTAATAAGTC-3′ and DyNAzyme DNA polymerase (Finnzymes) in order to amplify head-to-tail-ligated intron 1-containing *nad2* transcripts. PCR reactions were analysed on 3% (w/v) agarose gels, and products of interest were excised, purified over NucleoSpin columns (Macherey-Nagel), and ligated into pGEM-T (Promega) for cloning and sequencing.

### Analysis of excised introns by cRT-PCR/RT-PCR

RNA (1 μg) was treated with T4 RNA ligase (New England Biolabs) or mock-treated, extracted and precipitated as detailed above and dissolved in water. The RNA was then reverse-transcribed using gene-specific primers and SuperScript II Reverse Transcriptase (Life Technologies). The products of reverse transcription were again extracted and precipitated and subsequently amplified by PCR, using 1/10 of the RT reaction as template, intron-specifc forward and reverse primers and DyNAzyme DNA polymerase (Finnzymes). *nad5* intron 3-derived products were amplified with Phusion DNA polymerase (Finnzymes). PCR reactions were analysed on 3% (w/v) agarose gels. Products of interest were excised, purified over NucleoSpin columns (Macherey-Nagel) and ligated into pGEM-T (Promega) for cloning and sequencing. Several clones were sequenced per intron. A list of all gene-specific primers used in reverse transcription and PCR reactions is provided in Supplementary Table S3.

## RESULTS

### ODB1 deficiency alters the *nad2* transcript pattern

**Figure 1.**
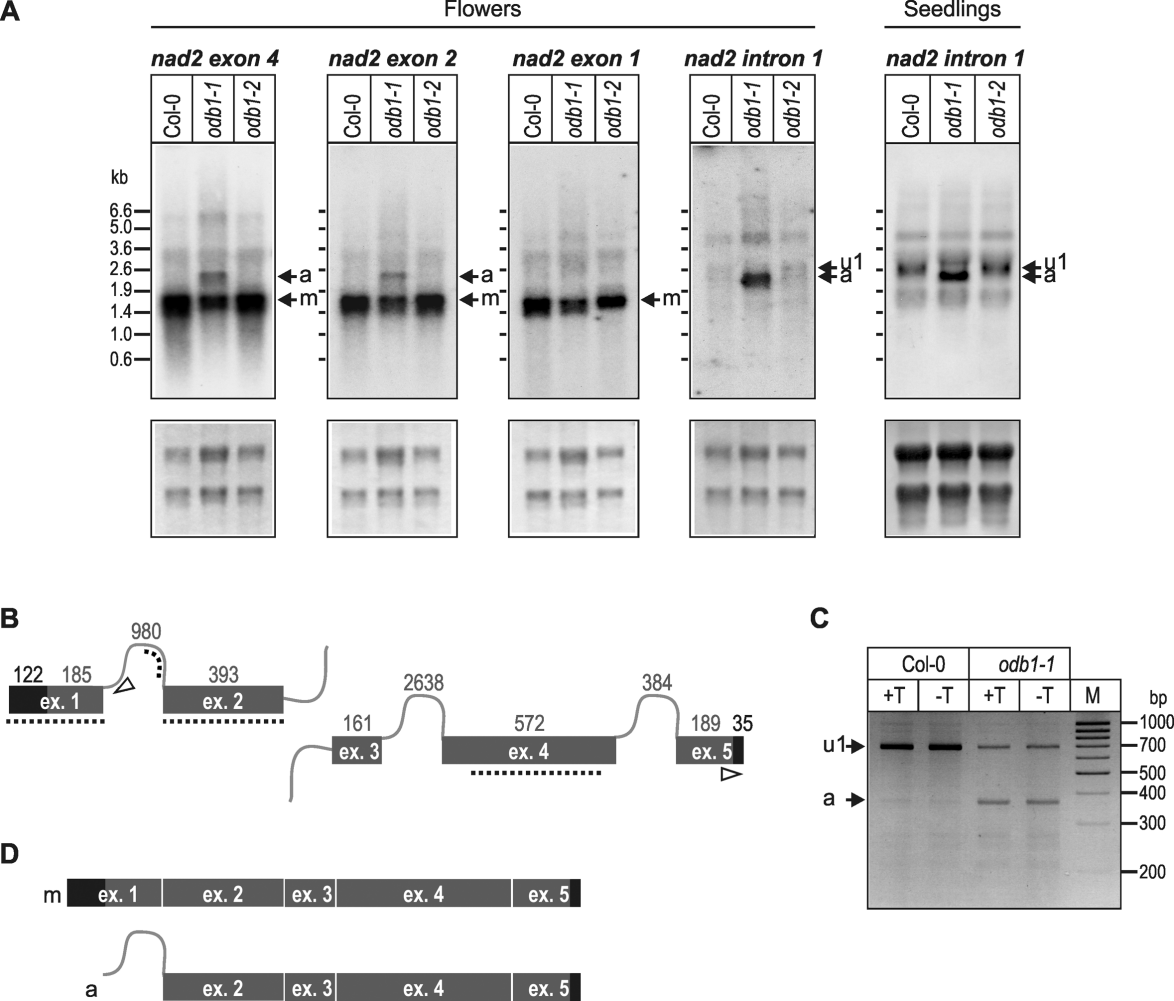
Incompletely spliced *nad2* transcripts over-accumulate in Arabidopsis plants lacking ODB1. (**A**) Probes for exon 4, exon 2, exon 1 and intron 1 of the mitochondrial *nad2* gene were hybridised to filter-immobilised total RNA isolated from *odb1–1, odb1–2* and wild-type (Col-0) flowers and seedlings (top panels). RNA marker sizes are indicated. Signals corresponding to the mature *nad2* mRNA (m), an alternative transcript of about 2300 nt over-accumulating in *odb1–1* (a) and transcripts from which the first intron has not been removed (u1) are indicated by arrows. The same filters stained with methylene blue are shown as loading controls in the bottom panels. (**B**) The *nad2* gene has five exons that are transcribed from two mtDNA regions and joined by *trans*-splicing of intron 2. Lengths of exons and *cis*-spliced introns are given in nucleotides. Only exons are drawn to scale (grey, coding regions; black, untranslated regions); introns are depicted as thin grey lines. Dotted lines mark probe target regions. Arrowheads indicate positions of primers used in the cRT-PCR experiment shown in (C). (**C**) The 5′ end of the *nad2* transcript over-accumulating in *odb1–1* was identified by cRT-PCR performed on TAP-treated (+T) and untreated (−T) seedling RNA. Products obtained for *odb1–1* and the wild type (Col-0) were separated on agarose gels alongside a molecular weight marker (M), cloned and sequenced. A product seen for both genotypes corresponded to *nad2* transcripts retaining the first intron (u1). A smaller product preferentially amplified from *odb1–1* (a) corresponded to a *nad2* transcript retaining intron 1 but lacking exon 1. With a length of 2330 nt this transcript matches the size of the *odb1–1*-specific transcript seen in RNA gel blots in (A). Amplification of the products labelled a and u1 strictly depended on the RNA ligation step during the cRT-PCR procedure (Supplementary Figure S1). (**D**) Schematic illustrating the structure of the *nad2* transcript over-accumulating in *odb1–1* (a) compared with the mature *nad2* mRNA (m).

We recently identified the RAD52-like protein ODB1 as a component of homologous recombination (HR)-dependent DNA repair in mitochondria of Arabidopsis (37). When subsequently examining the impact of ODB1 loss on mitochondrial gene expression, we detected an alternative *nad2* transcript that over-accumulated in *odb1–1* mutant plants but was barely seen in the wild type or in *odb1–2* (Figure 1A). *odb1–2* plants had been found earlier to express reduced amounts of ODB1 protein; in contrast, *odb1–1* plants were considered complete loss-of-function mutants, due to lack of detectable levels of ODB1 (37). *nad2* in Arabidopsis is transcribed from two different mitochondrial genome (mtDNA) regions. Maturation of its mRNA requires one *trans*- and three *cis*-splicing events (41)(Figure 1B). To determine possible defects in mtDNA maintenance or gene expression responsible for the altered *nad2* transcript pattern in *odb1–1*, we hybridised RNA gel blots with probes specific for different parts of the *nad2* transcript. The *odb1–1*-specific *nad2* transcript was detected in both seedlings and flowers using probes for exons 2 or 4 but not exon 1 (Figure 1A). A transcript of the same size was detected in *odb1–1* using a probe annealing to intron 1, indicating that *odb1–1* over-accumulated a *nad2* transcript containing at least part of intron 1 but lacking exon 1. The amount of mature *nad2* mRNA was slightly reduced in *odb1–1* but not *odb1–2* compared with the wild type, suggesting that over-accumulation of the alternative *nad2* transcript in *odb1–1* was at the expense of mature *nad2* mRNA formation.

### ODB1 is required for the efficient excision of intron 1 from *nad2* pre-mRNAs

We assumed that the *odb1–1*-specific *nad2* transcript resulted either from incorrect transcript maturation or from previously undetected mtDNA recombination in this mutant and expression of the *nad2* 5′-portion from an alternative mtDNA region lacking exon 1. To distinguish between these possibilities and determine the 5′ end of the *odb1–1*-specific transcript, RT-PCR was performed on head-to-tail-ligated RNA (cRT-PCR) for both *odb1–1* and wild-type seedlings. We ensured that a potential alternative primary *nad2* transcript for *nad2* would also be ligated by pre-treating RNA samples with tobacco acid pyrophosphatase (TAP). TAP converts primary, 5′-triphosphorylated mitochondrial transcripts into 5′-monophosphorylated transcripts (40). Only the latter are substrates for T4 RNA ligase (42). A cRT-PCR product was amplified from *odb1–1* that was hardly detectable in the wild type (Figure 1C). Amplification of this product was not dependent on or enhanced by TAP treatment, implying it was derived from a 5′-processed RNA. Subsequent cloning and sequencing showed that this product corresponded to the previously mapped *nad2* 3′ end (43) fused to the 5′-end of intron 1 (Supplementary Table S4). The alternative transcript over-accumulating in *odb1–1* accordingly contained the complete intron 1 but lacked exon 1 (Figure 1D). We thus concluded that (i) ODB1 is required for the efficient removal of the *cis*-spliced intron 1 from *nad2* and (ii) excision of this intron may proceed via first-step hydrolysis rather than lariat formation in Arabidopsis.

### ODB1 additionally stimulates splicing of *nad1* intron 2

**Figure 2.**
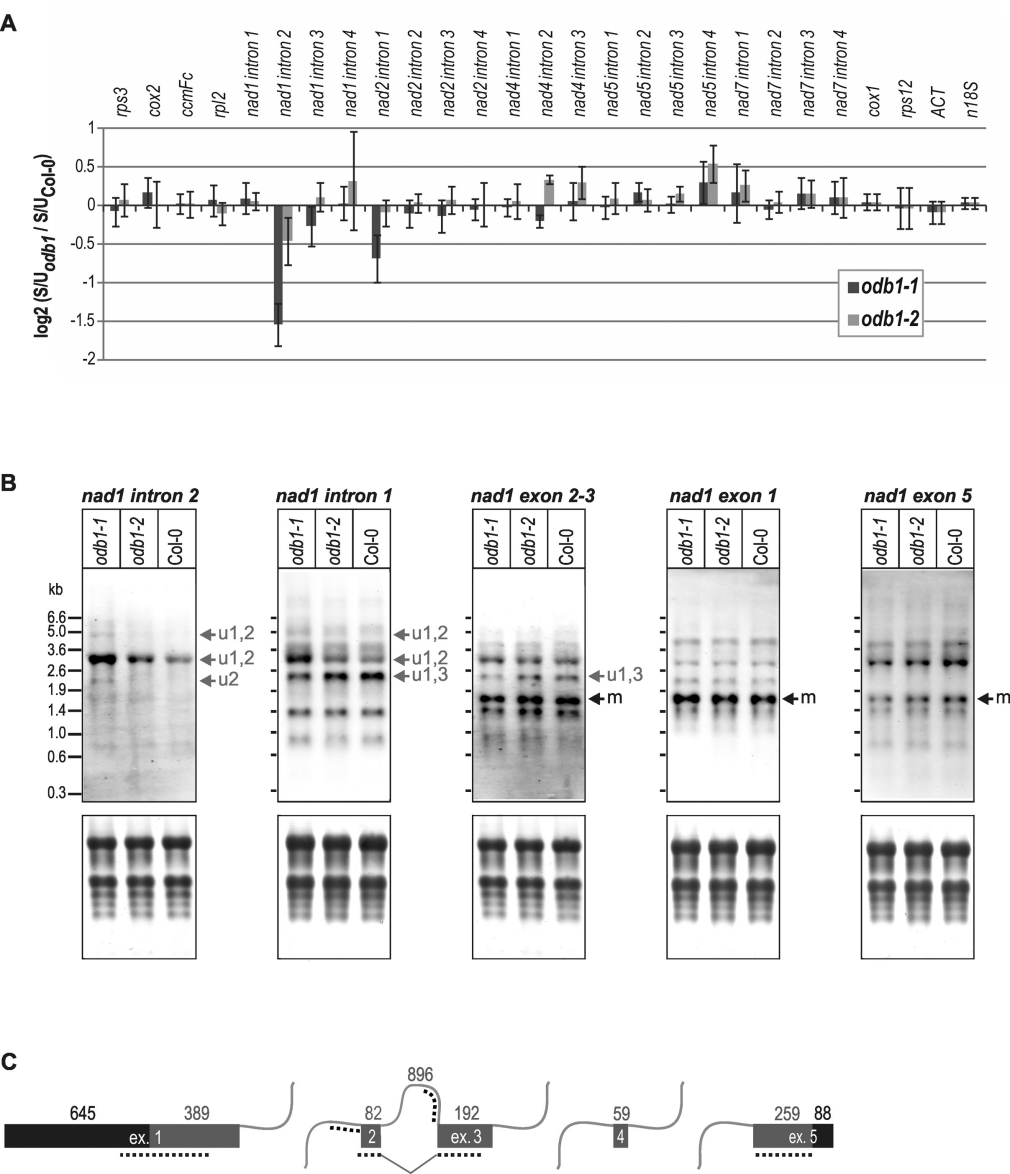
Altered *nad1* mRNA splicing in *odb1*. (**A**) Mitochondrial transcript splicing efficiencies were compared between the wild type (Col-0), *odb1–1* and *odb1–2* for all 23 mitochondrial introns, using a previously described qRT-PCR assay (16,22). This assay quantifies spliced transcripts by amplification of products that span splice junctions and unspliced transcripts by amplification of products that span exon-intron junctions. The histogram depicts splicing efficiencies as the ratio of spliced to unspliced products amplified from the mutant divided by the ratio of spliced to unspliced products amplified from the wild type, using a log_2_ scale. Three technical replicates of two independent biological repeats were performed for mutant and wild-type plants for each transcript; standard errors are indicated. Transcript levels were additionally analysed for two intron-less mitochondrial genes (*cox1, rps12*); the nuclear *18S rRNA* and *ACT* genes were used for data normalisation. (**B**) Probes for intron 1, intron 2, exon1, exons 2 and 3 (intron-spanning probe), and exon 5 of the mitochondrial *nad1* gene were hybridised to filter-immobilised total RNA isolated from *odb1–1, odb1–2* and wild-type (Col-0) seedlings (top panels). RNA marker sizes are indicated. Prior to hybridisations, membrane-bound RNAs were stained with methylene blue (bottom panels). Signals for the mature *nad1* mRNA are indicated (m). Both *odb1–1* and *odb1–2* over-accumulate *nad1* transcripts with introns 1 and 2 unspliced (u1,2). The exact composition of these transcripts is unclear as they were not detected with any of the exon-specific probes. The same holds true for a transcript of low abundance detected only in *odb1–1* with the intron 2-specific probe (u2). The signal for this RNA was more pronounced in a similar experiment (Supplementary Figure S2). A transcript exclusively detected with probes for intron 1 and exons 2 and 3 and thus lacking exons 1 and 5 is reduced in *odb1–1* (u1,3). This transcript also lacks exon 4 since no transcript of this size was detected with an exon 4-specific probe in an earlier study (58). (**C**) The *nad1* gene has five exons that are joined by one *cis*- and three *trans*-splicing events. Lengths of exons and the *cis*-spliced intron are given in nucleotides; symbols are as in Figure 1. Dotted lines mark probe target regions.

To examine whether ODB1 has roles in splicing of further mitochondrial transcripts, we applied a qRT-PCR assay designed for monitoring splicing efficiencies for all 23 mitochondrial introns in Arabidopsis (16,22). The genes *cox2, ccmFc, rpl2* and *rps3* each contain a single intron, *nad4* contains three introns and *nad1, nad2, nad5* and *nad7* each contain four introns (41). While the qRT-PCR assay barely detected the *nad2* splicing defect in *odb1–1*, it revealed that excision of the *cis*-spliced intron 2 from *nad1* pre-mRNAs was compromised in *odb1–1* (Figure 2A). Over-accumulation of transcripts containing this intron in *odb1–1* was confirmed by RNA gel blot hybridisation with an intron 2-specific probe (Figure 2B). A mild splicing defect was also seen in plants homozygous for the hypomorphic *odb1–2* allele. Removal of *nad1* intron 2 and *nad2* intron 1 in *odb1–1* was impaired at different steps. For incompletely processed *nad2*, hydrolysis occurred at the 5′ end of intron 1 but splicing was arrested after this step, leading to the presence of the intron and absence of exon 1. For incompletely processed *nad1*, intron 2 splicing was not initiated, and mRNAs that were overrepresented in *odb1–1* had a fully inserted intron 2. RNA gel blot hybridisation with a probe specific for the intron 1 3′-portion showed that the major *nad1* transcript species which over-accumulated in *odb1–1* contained both intron 2 and the 3′ portion of the trans-spliced intron 1, which is co-transcribed with exons 2 and 3. No transcripts were elevated in *odb1–1* from which intron 2 had been excised but that still contained the 3′ part of intron 1. Hence, altered intron 1 splicing in *odb1–1* was not a direct consequence of ODB1 absence but linked to impaired intron 2 splicing. Reduced trans-splicing of an intron as a consequence of impaired cis-splicing of a downstream intron has also been observed in the *rug3* mutant lacking a protein involved in the removal of the third intron from *nad2* pre-mRNAs (22). Hybridisations with exon-specific probes revealed no reduction in the mature *nad1* mRNA in *odb1–1* (Figure 2B). However, a transcript with processed intron 2 but unprocessed intron 1 (labelled u1,3 in Figure 2B) was reduced in *odb1–1*. This indicates that over-accumulation of *nad1* transcripts containing intron 2 in *odb1–1* was at the expense of transcripts with processed intron 2, substantiating a role for ODB1 in *nad1* intron 2 splicing. Secondly, it may in part explain why qRT-PCR experiments did not detect reduced *nad1* intron 1 splicing in *odb1–1* despite the over-accumulation of some intron 1-containing *nad1* transcripts, and it supports that *odb1–1* plants have no primary defect in *nad1* intron 1 splicing.

We performed RNA gel blot hybridisations with probes for most other mitochondrial introns and found that in agreement with qRT-PCR data, splicing of mitochondrial introns other than *nad2* intron 1 and *nad1* intron 2 was not affected in *odb1–1* mutants (Supplementary Figure S2). Introduction of an *ODB1* wild-type gene copy into *odb1–1* plants restored ODB1 protein expression and efficient excision of both *nad2* intron 1 and *nad1* intron 2 (Supplementary Figure S3), confirming that reduced splicing of these introns in *odb1–1* plants was due to ODB1 loss. ODB1 is thus required for efficient splicing of two mitochondrial introns, *nad1* intron 2 and *nad2* intron 1, *in vivo*.

### ODB1-dependent introns are two out of several hydrolytically spliced introns in mitochondria

**Figure 3.**
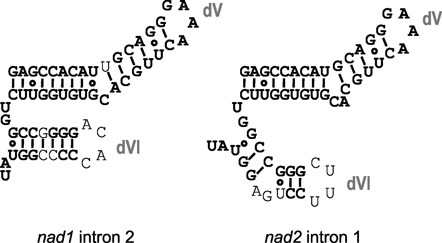
Secondary structure models of domains dV and dVI of *nad1* intron 2 and *nad2* intron 1 from Arabidopsis, as previously derived (34). Bold nucleotides are identical between the two intron sequences.

To further elucidate the role of ODB1 in mitochondrial transcript splicing we investigated common properties of the two introns whose excision was affected in *odb1* mutants. Both introns are classified as group II introns (35). Aligning their sequences showed 86% sequence identity in structural domains dV and dVI (Figure 3); neither intron displayed a comparable degree of identity with any other mitochondrial intron in these domains according to BLAST searches (http://blast.ncbi.nlm.nih.gov). Sequence identity between *nad1* intron 2 and *nad2* intron 1 in domains dV and dVI as well as in other domains and the intron structural core has been previously noted in the genus *Oenothera*, and a common evolutionary origin of the two introns has been proposed (44). dV and dVI are important for the group II intron splicing mechanism, with dVI usually containing an unpaired, bulged adenosine that acts as attacking nucleophile during the first step of splicing and marks the branch point in introns liberated as lariats (3,27).

In addition to showing a high degree of sequence identity, *nad1* intron 2 and *nad2* intron 1 have in common that they differ from the group II intron consensus. According to previously derived secondary structure models (33,34), *nad1* intron 2 completely lacks a bulged nucleotide in dVI, with the latter forming a tight helix (Figure 3). Within the *nad2* intron 1 dVI helix, an unpaired adenosine is preceded by an unpaired guanosine; these nucleotides are flanked by Watson-Crick or G:U wobble base pairs (Figure 3). Whereas introns with a similar, non-conventional dVI structure were previously shown to splice as lariats in higher plant chloroplasts (32), the nature of incompletely spliced *nad2* transcripts over-accumulating in *odb1–1* (Figure 1) indicated hydrolytic excision of *nad2* intron 1 in mitochondria of Arabidopsis. In wheat mitochondria, both *nad1* intron 2 and *nad2* intron 1 lack a dVI bulged adenosine and use a hydrolytic pathway (33).

To test for hydrolytic excision of *nad1* intron 2 and *nad2* intron 1 in Arabidopsis we applied a previously described cRT-PCR/RT-PCR strategy for determining the structure of released introns (32,34,45) (Supplementary Figure S4). Briefly, intron extremities are amplified using a forward primer annealing to the intron 3′-region upstream of dVI and a reverse primer annealing to the intron 5′-part, followed by cloning and sequencing of products. For introns released as lariats, this will yield molecules in which the intron 5′ and 3′ ends are joined at the branch point but that lack the short lariat 3′-tail sequence of the intron. In contrast, amplification of introns that are hydrolytically spliced and released as linear molecules will require intron head-to-tail ligation prior to RT-PCR; products will contain the complete intron 3′-sequence. For hydrolytically spliced introns released as circular molecules, products will also contain the complete intron 3′-end but will not depend on intron ligation.

**Figure 4.**
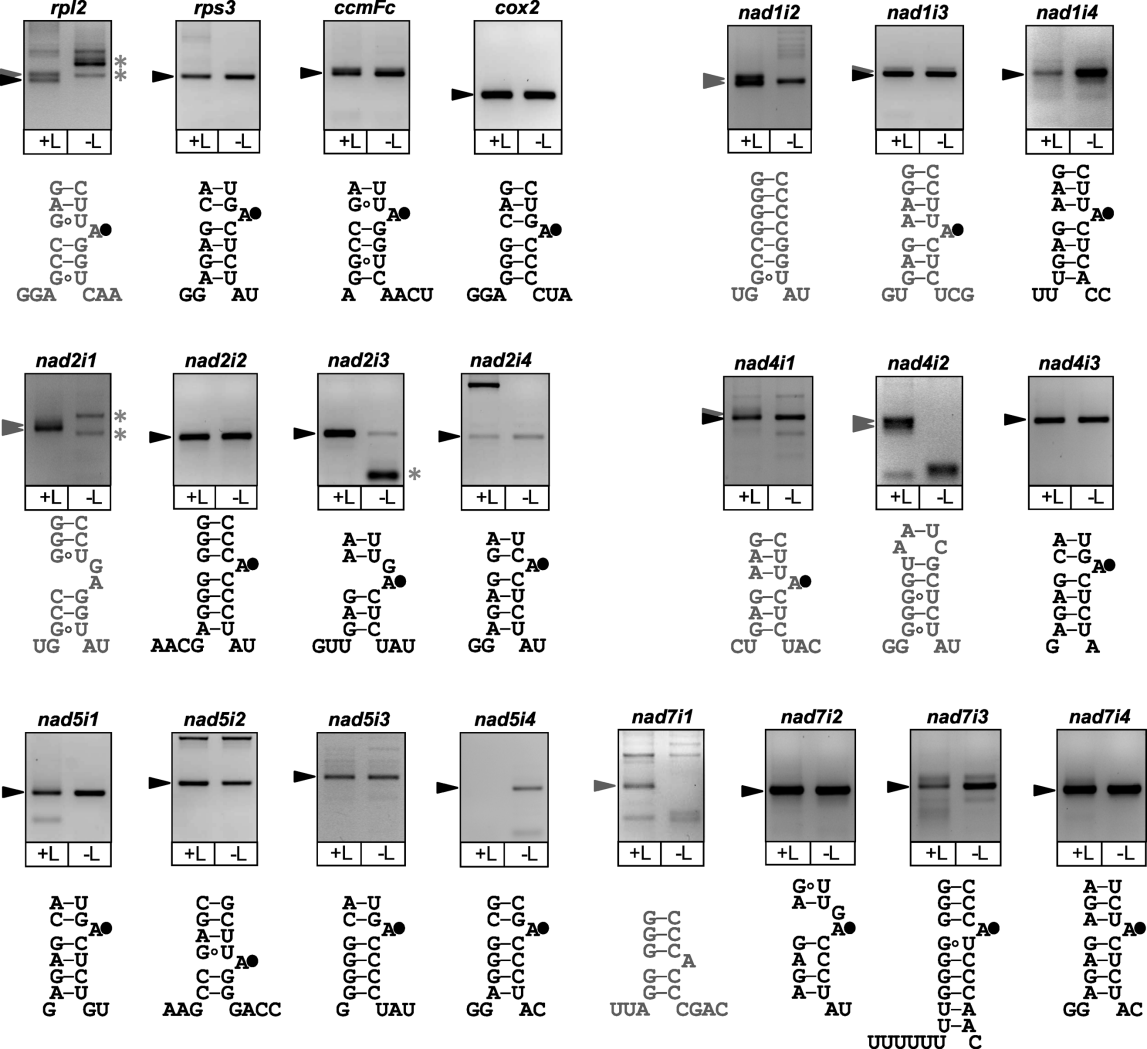
Analysis of Arabidopsis mitochondrial group II intron splicing pathways. The structures of released mitochondrial introns were analysed by RT-PCR performed on ligase-treated (lanes +L, cRT-PCR) and untreated (-L, RT-PCR) RNA extracted from Arabidopsis wild-type seedlings, using primers annealing to intron sequences as illustrated in Supplementary Figure S4. *nad1* intron 2 (*nad1i2*; other *nad* gene introns are named accordingly) and *nad2* intron 1 were additionally analysed for the *odb1–1* mutant; results for these experiments are displayed in Supplementary Table S5. Gel images show products separated on 3% (w/v) agarose gels. Following cloning and sequencing of cRT-PCR and RT-PCR fragments, products labelled with black and grey arrowheads were identified to derive from introns released via lariat formation and first-step hydrolysis, respectively. Asterisks mark products that were found to be non-specific. dV lower stem models are shown below each gel image for the respective mitochondrial intron. To derive these models, homologous intron sequences from different plant species were aligned, putative dV regions were identified by folding conserved parts of the intron 3′ region (not displayed), and dVI stem-loops were then modelled from sequences downstream to dV. The mfold web server (59) was used for deriving putative dV and dVI secondary structures. Secondary structure models have previously been proposed for the four Arabidopsis *nad5* introns, (60), *nad7* intron 3 (61), *nad1* intron 2, *nad2* intron 1, *nad2* intron 4 and *nad4* intron 2 (34); they are identical with those derived here. dVI stems of introns that were found here to exclusively splice via the lariat pathway are depicted in black, and unpaired dVI nucleotides identified experimentally as lariat branch point are labelled with a filled black circle. dVI stem models of introns for which the use of a hydrolytic pathway was detected are depicted in grey. Grey stem models with unpaired nucleotides marked with a filled black circle correspond to introns for which the use of both a hydrolytic pathway and the lariat pathway was observed. Note that *nad5* intron 4, which splices via the lariat pathway, failed to yield products from ligated RNA.

For *nad1* intron 2, different products were amplified and sequenced that comprised the full intron 3′-sequence and thus corresponded to introns released via first-step hydrolysis (Figure 4; see Supplementary Table S5 for sequence analyses). Amplification from ligated RNA produced two fragments of similar size, the smaller corresponding to the head-to-tail ligated intron and the larger containing an additional stretch of non-encoded adenosines and cytidines at the ligation site. The former was also amplified from non-ligated RNA, indicating that a fraction of the released intron molecules persist in a circular form. Excised circular intron molecules have been previously detected in plant mitochondria; they were proposed to derive from in vivo circularisation of linear excised introns (34) or intron release in a circular form (46). The stretches of non-encoded adenosines and cytidines seen in ligation-derived sequences have been proposed to be added to excised linear introns in order to tag these molecules for the bacterial-type RNA degradation pathway in mitochondria (33). In the course of our study they were detected for several other introns spliced via first-step hydrolysis (see below, Supplementary Table S5). None of the products obtained for *nad2* intron 1 supported conventional lariat formation despite the presence of a dVI unpaired adenosine; sequenced molecules were indicative of a hydrolytic pathway and linear intron release (Figure 4 and Supplementary Table S5). It should be noted that lariats, which contain a 2’-5′-phosphodiester bond at the branching point, are suboptimal substrates for reverse transcription. Failure to detect lariat molecules by RT-PCR does therefore not exclude their occurrence. Released *nad1* intron 2 and *nad2* intron 1 did not differ between *odb1–1* and wild-type Arabidopsis plants (Supplementary Table S5).

We subsequently applied the cRT-PCR/RT-PCR strategy to all mitochondrial introns in order to systematically determine splicing pathways used in Arabidopsis mitochondria (Figure 4 and Supplementary Table S5) and assess whether ODB1 might specifically stimulate the splicing of introns depending on first-step hydrolysis. dVI base-pairing models predict that like *nad1* intron 2, *nad4* intron 2 and *nad1* intron 1 lack an unpaired adenosine whereas this potential lariat branching point is present in all other Arabidopsis mitochondrial introns (Figure 4). The latter intron, which was the only intron for which we failed to obtain cRT-PCR/RT-PCR products, is not included in Figure 4. A dVI model for this highly unusual intron has been previously proposed that is indicative of a non-lariat pathway (34).

**Figure 5.**
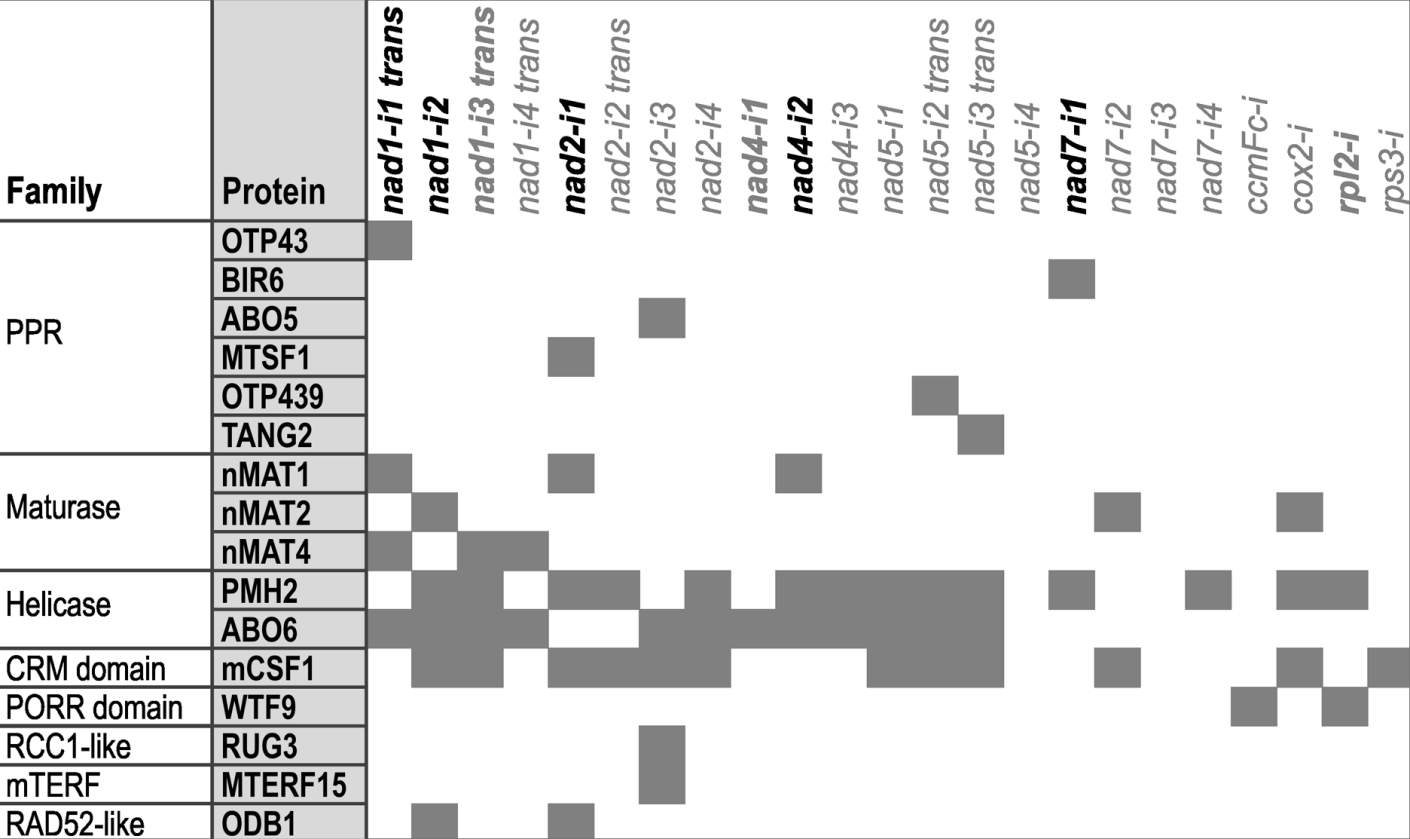
Factors that promote group II intron splicing in Arabidopsis mitochondria. The diagram lists protein families and their members whose role in mitochondrial group II intron splicing has been inferred from analyses of loss-of-function mutants (see introduction for references) and indicates which introns depend on the function of a specific factor. Introns are named as in Figure 4; *trans*-spliced introns are indicated. Bold black introns are spliced exclusively via first-step hydrolysis, bold grey introns use both lariat and hydrolytic pathways, and all other introns are released as lariats. For *nad1* intron 1, a hydrolytic pathway is assumed based on its secondary structure model (34).

In addition to *nad1* intron 2 and *nad2* intron 1, two other introns, *nad4* intron 2 and *nad7* intron 1, did not yield lariat-derived RT-PCR products and appear to exclusively use a hydrolytic pathway. For *nad1* intron 3, *nad4* intron 1 and the single *rpl2* intron, hydrolytically released molecules as well as lariats were detected; all other introns showed lariat-derived products. According to these results, ODB1 is not generally required for efficient release of hydrolytically spliced introns but has a specific role in the excision of *nad1* intron 2 and *nad2* intron 1. Combining our analysis of splicing pathways with current data on the specificities of known mitochondrial splicing factors shows that none of these factors is common and exclusive to introns released via a hydrolytic pathway (Figure 5).

## DISCUSSION

Transcript splicing in higher plant organelles involves numerous ancillary factors most of which can be classified as intron maturases, RNA helicases or members of various RNA-binding protein families (4,5). A number of recent studies indicate that plant organelles moreover recruited several proteins into their splicing machineries that belong to protein families initially characterised to perform unrelated functions. One of these proteins is the RCC1 family protein RUG3 required for splicing of the *nad2* mRNA in Arabidopsis mitochondria (22). The maize Zm-MTERF4 protein and its Arabidopsis orthologue BSM involved in chloroplast group II intron splicing (25,47) as well as the recently identified mitochondrial splice factor mTERF15 (23) belong to the mTERF family whose members in animal mitochondria have roles in transcription and translation (48–50). The family of WHIRLY proteins initially characterised as nuclear transcription factors (51) and shown later to contribute to genome stability in plastids and mitochondria (52,53) includes maize WHY1 required for the splicing of several introns in chloroplasts (54). This protein, which bound DNA without sequence specificity, was additionally found to specifically associate with introns whose splicing it facilitated (54).

Results presented here demonstrate a role for the protein ODB1 in group-II intron splicing in mitochondria of Arabidopsis. ODB1-deficient plants displayed reduced splicing efficiencies for *nad1* intron 2 and *nad2* intron 1, both of which were found to splice via a hydrolytic pathway. ODB1 is distantly related to RAD52 (37), a protein involved in homology-dependent DNA repair and recombination in the nucleus (55). Accordingly, ODB1 and its plastid paralogue ODB2 described earlier as RAD52–1 and RAD52–2, respectively, have been reported to function in nuclear DNA repair (38). However, both proteins predominantly localise to mitochondria and plastids (37,38), and ODB1 was later identified as a component of homologous recombination (HR)-dependent DNA repair in mitochondria (37). The dual function of ODB1 as a DNA repair component and a splice factor in Arabidopsis mitochondria is reminiscent of maize WHY1 and adds another multifunctional protein to the list of organellar splice factors. The role of ODB2 in plastids is as yet unknown.

ODB1 is assumed to be rather abundant in Arabidopsis mitochondria (56). Interestingly, while *odb1–1* plants which completely lacked ODB1 displayed defects in both HR-dependent DNA repair and *nad1* and *nad2* splicing, *odb1–2* plants with strongly reduced but detectable amounts of ODB1 protein were impaired in DNA repair to a degree that was comparable to *odb1–1* but showed no splicing defect for *nad2* intron 1 and accumulated significantly less incompletely spliced *nad1* transcripts than *odb1–1* (37 and this work). This implies that the bulk of mitochondrial ODB1 is likely required for mitochondrial genome maintenance, presumably to cover large stretches of single-stranded DNA, and only a small fraction of mitochondrial ODB1 is involved in splicing. The accumulation of significant amounts of correctly processed *nad1* and *nad2* in *odb1–1* shows that ODB1 is not strictly required for the splicing of these transcripts but has an accessory role. Participation of ODB1 in splicing may be more crucial under conditions that reduce splicing efficiencies, e.g. during growth in the cold (33). ODB1 could facilitate splicing by stabilising the structure of correctly folded *nad1* intron 2 and *nad2* intron 1 in domains dV and dVI, which are highly similar between the two introns, either through interaction with other components of the splicing machinery or by direct intron binding. Organellar proteins that bind both RNA and DNA have been previously described (54,57).

In the course of this study we performed the first systematic analysis of mitochondrial group II intron splicing pathways, which underscored unusual aspects of splicing in plant mitochondria. Although excision of both *nad1* intron 2 and *nad2* intron 1 depends on first-step hydrolysis it is unlikely that ODB1 is a general determinant of the hydrolytic pathway. Two other introns, *nad4* intron 2 and *nad7* intron 1, were identified that spliced exclusively via a hydrolytic pathway in Arabidopsis but did not depend on ODB1. In addition, *nad1* intron 1 is likely hydrolytically released (34) but its splicing was not directly affected by *ODB1* gene inactivation. It is nevertheless expected that *trans*-acting factors impact on the biochemistry of mitochondrial group-II intron splicing. *nad7* intron 1, which in dVI is predicted to have a bulged A flanked by G:C pairs and was thus expected to be released as lariat, appears to exclusively splice via first-step hydrolysis. On the other hand, *nad2* intron 3 and *nad7* intron 2 which have predicted dVI configurations similar to *nad2* intron 1 were found to strictly use the lariat pathway. It thus seems that intron folding and the presence/absence of a dVI unpaired adenosine do not exclusively define the group II intron splicing pathway in higher plant mitochondria, and other factors probably influence whether intron excision proceeds via first-step transesterification or hydrolysis. As yet there is no candidate splice factor that could potentially determine the biochemistry of intron release as none of the mitochondrial splice factors identified to date is common and exclusive to introns that splice via a hydrolytic pathway.

## SUPPLEMENTARY DATA

Supplementary Data are available at NAR Online.

SUPPLEMENTARY DATA
