# Supplementary material for: The RAD52-like protein ODB1 is required for the efficient excision of two mitochondrial introns spliced via first-step hydrolysis
Source: Nucleic Acids Res. 2015 Jun 5;43(13):6500–10. doi: 10.1093/nar/gkv540 (PMC4513849; doi:10.1093/nar/gkv540)
Supplement: SUPPLEMENTARY DATA [file supp_43_13_6500__index.html]

The RAD52-like protein ODB1 is required for the efficient excision of two mitochondrial introns spliced via first-step hydrolysis — The RAD52-like protein ODB1 is required for the efficient excision of two mitochondrial introns spliced via first-step hydrolysis — SUPPLEMENTARY DATA 

# The RAD52-like protein ODB1 is required for the efficient excision of two mitochondrial introns spliced via first-step hydrolysis

## SUPPLEMENTARY DATA

- SUPPLEMENTARY DATA
