# Supplementary material for: The RAD52-like protein ODB1 is required for the efficient excision of two mitochondrial introns spliced via first-step hydrolysis
Source: Nucleic Acids Res. 2015 Jun 5;43(13):6500–10. doi: 10.1093/nar/gkv540 (PMC4513849; doi:10.1093/nar/gkv540)
Supplement: SUPPLEMENTARY DATA [file supp_gkv540_nar-00175-a-2015-File007.pdf]

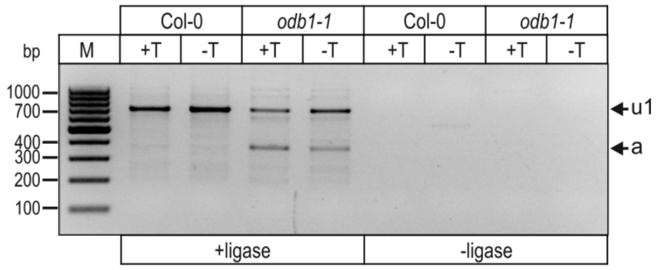

Supplementary Figure S1. Amplification of cRT-PCR products a and u1 as shown in Figure 1C depends on ligase treatment of transcripts. cRT-PCR assays were performed as in Figure 1C, except that control experiments were simultaneously performed in which no T4 RNA ligase was added during the ligation step (- ligase lanes). See Figure 1 legend for further details.

A

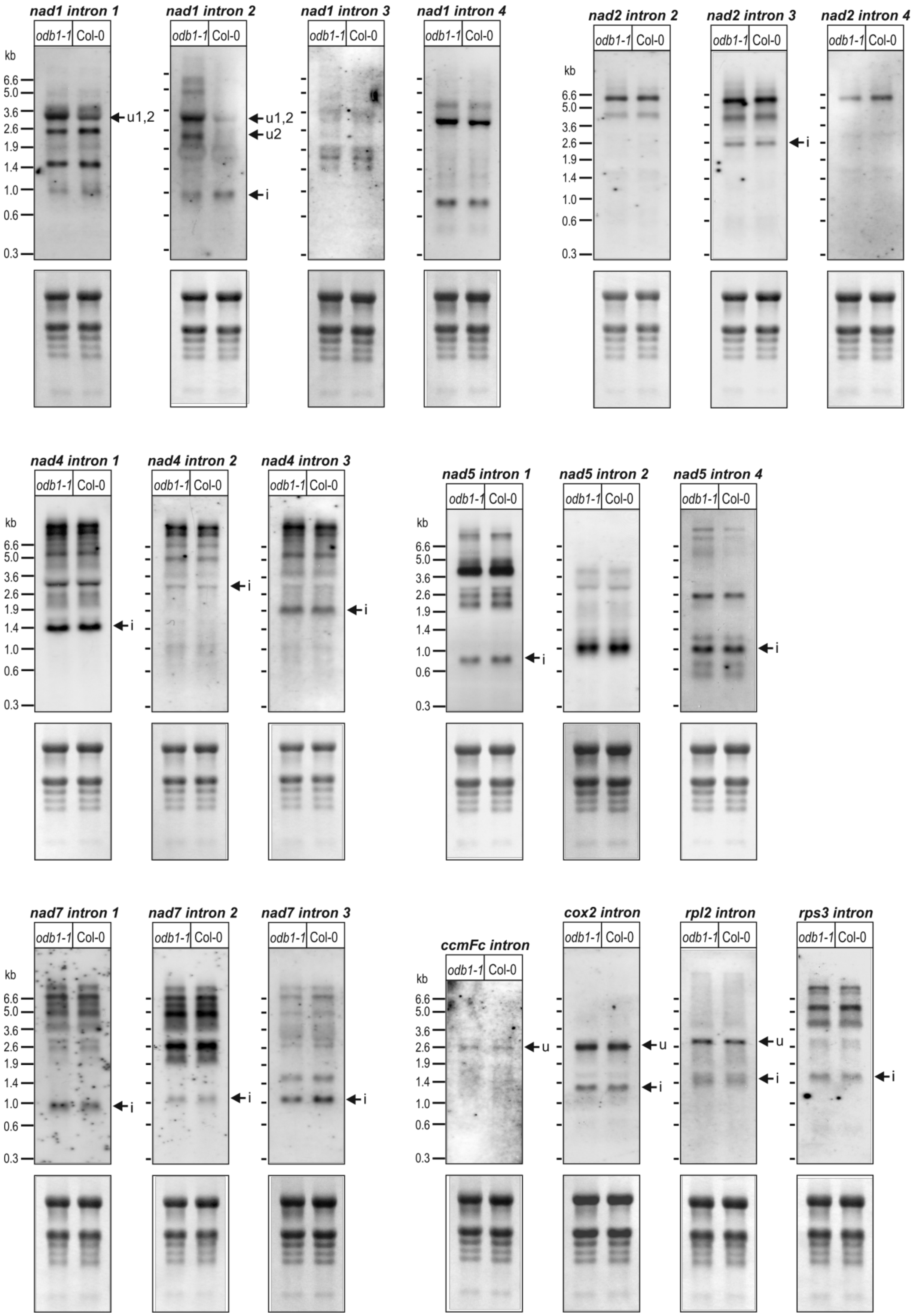

nad1 intron 1
nad1 intron 2
nad1 intron 3
nad1 intron 4
nad2 intron 2
nad2 intron 3
nad2 intron 4
nad4 intron 1
nad4 intron 2
nad4 intron 3
nad5 intron 1
nad5 intron 2
nad5 intron 4
nad7 intron 1
nad7 intron 2
nad7 intron 3
ccmFc intron
cox2 intron
rpl2 intron
rps3 intron
odb1-1
Col-0
kb
6.6
5.0
3.6
2.6
1.9
1.4
1.0
0.6
0.3
u1,2
u2
u
i

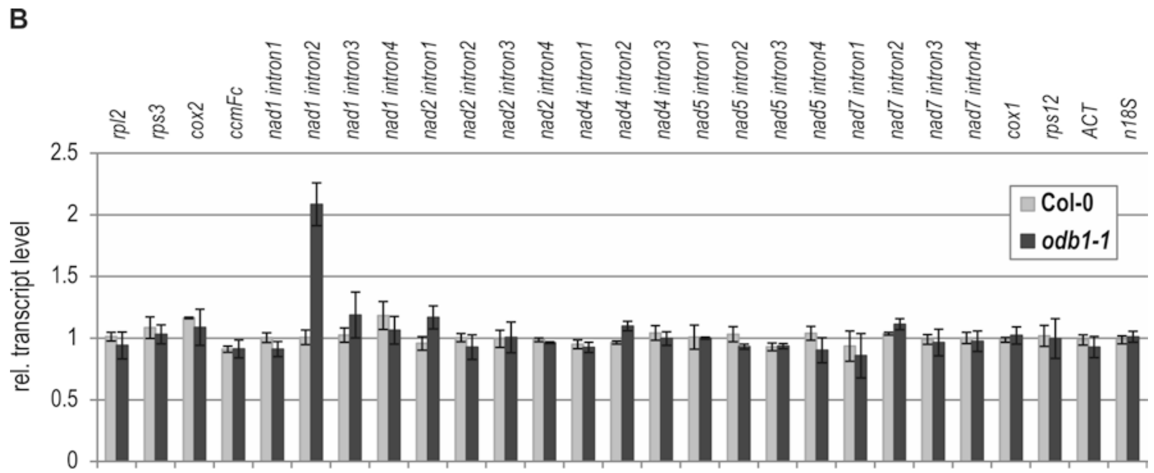

Supplementary Figure S2. The splicing of introns other than *nad2* intron 1 and *nad1* intron 2 is not affected by *ODB1* inactivation. (A) To assay the *odb1-1* mutant for over-accumulating unspliced mitochondrial transcripts, probes for 20 out of the 23 mitochondrial introns were hybridised to filter-immobilised total RNA isolated from *odb1-1* and wild-type (Col-0) seedlings (top panels). RNA marker sizes are indicated. Selected transcripts are marked that were inferred from their sizes as being released introns (i) or unspliced transcripts (u); see Figure 2 for *nad1* transcript labels. The methylene blue-stained filters are shown as a loading control in the bottom panels. Blots for *nad5* intron 3 and *nad7* intron 4, for which no signals were detected, are not displayed; hybridisations with *nad2* intron 1 probes are shown in Figure 1. (B) Transcripts that are unspliced for a specific intron were measured by qRT-PCR, using primer pairs amplifying across intron-exon borders. Median-normalised relative transcripts levels and standard errors are shown. Intron-free mitochondrial transcripts *cox1* and *rps12* and nuclear transcripts *ACT* and *18S* were included as controls. Surprisingly, over-accumulation of transcripts retaining *nad2* intron 1 in *odb1-1* is not apparent from this analysis.

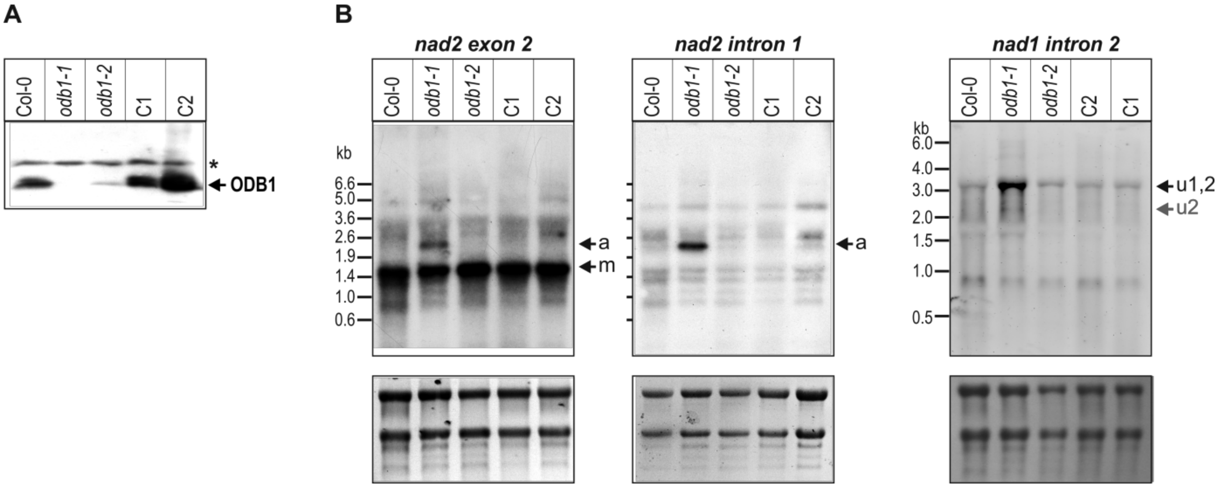

Supplementary Figure S3. Complementation of *odb1-1* mutants with a functional *ODB1* gene copy restores wild type-like splicing of *nad1* and *nad2* transcripts. (**A**) An ODB1 antibody (37) was used to probe immunoblots of leaf extracts prepared from wild-type, *odb1-1*, *odb1-2* plants and two independent complemented plant lines (C1, C2). Signals corresponding to ODB1 are indicated; a signal marked with an asterisk might be due to a cross-reaction of the antibody with chloroplastic ODB2. The complemented lines show wild type-like or higher levels of ODB1. (**B**) Probes for *nad2* exon 2 and intron 1 and for *nad1* intron 2 were hybridised to filter-immobilised total RNA isolated from *odb1-1*, *odb1-2* and wild-type (Col-0) flowers and from flowers of complemented lines C1 and C2 (top panels). RNA marker sizes are indicated. Hybridisation signals are labelled as in Figures 1 and 2. The complemented lines C1 and C2 displayed wild type-like transcript patterns, substantiating that the splicing defects seen in *odb1-1* were due to ODB1 loss. The methylene blue-stained filters are shown as a loading control in the bottom panels. The *nad1* intron 2 blot shown here does not indicate elevated unspliced transcripts in *odb1-2*; this is likely because less RNA was loaded for the *odb1-2* sample.

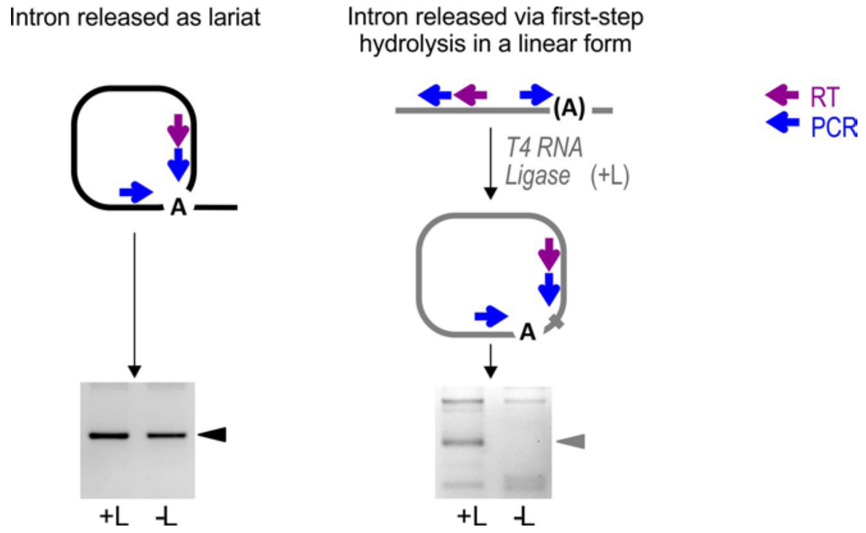

Supplementary Figure S4. Schematic illustrating the RT-PCR/cRT-PCR strategy applied to amplify branch-point regions and/or intron extremities in order to distinguish introns released via first-step hydrolysis (right) from those liberated as lariats (left). Positioning of primers used for reverse transcription (violet) and subsequent PCR (blue) is indicated. The method exploits the ability of reverse transcriptases to read across lariat branch points (45). Introns spliced via a hydrolytic pathway and released in a linear form (right) require head-to-tail ligation in order to yield PCR products. Sequences obtained for cDNAs made from these introns will contain the complete intron 3' sequence whereas sequences of lariat-derived cDNAs will lack nucleotides corresponding to the lariat tail. Sequences including the lariat tail but derived from introns that do not require ligation prior to amplification correspond to introns released via first-step hydrolysis that are subsequently circularised or to introns released in a circular form. Excised circular introns have been previously observed in maize mitochondria (34). Sequences of lariat-derived cDNAs will show a thymidine instead of an adenosine at the branch point (45).

Supplementary Table S1. Primers used for synthesizing DIG-labelled probes.

Probe	Primer 1	Primer 2
nad2 exon 1	CATTTTTTTATTGAGCCGAATCACT	TCCAAGCCAACCCACATTACTG
nad2 exon 2	CGGGCTTTCCTGAGGTATCTAA	GTAGTTGTCCGGTCGTACC
nad2 exon 4	CCGGTTACAGCATTCCTTTC	AGCGGCGAAGAACAAATAGA
nad2 intron 1	GGTGAGGAATATCCCGAGTCTC	CACACGTGCAAGTTTCCCTG
nad2 intron 2	AGTTAGAGAGGTTGGCGAACTACT	AAAGTACCTCTCCAATCCTCGAT
nad2 intron 3	GTTCTGATAAGGAAGGAACAACCC	TCTCTTTTCTAGTAGATGCCGAACC
nad2 intron 4	GGCAGAGGGTCCGTAGTAC	GATCAAAAAGGAAGTTTACTGGC
nad1 exon 1	CGTTCCATTGAGAATAGATGG	CGAGCGACCAGACTTAACAT
nad1 exon 2-3	GCTCGTACGGTTCATAGAAG	ACATTATAGCCTGCAACTGA
nad1 exon 5	CCATGCACATTGTTCTTTCCA	AAGGAAGCCATTGAAAGGTGACTA
nad1 intron 1	TGATTTTTTTGGCGGGTTC	GTTCAAGTTACCAACAATCAACGAG
nad1 intron 2	AAACCAGGGCAACAAATGTCG	AATGTGGCTCGTCCGTGCT
nad1 intron 3	CCCTGGATTCCCGGATTC	CAAGGCCATTTCTGTCCACC
nad1 intron 4	CGCTTGGACTTGTCACTTCGT	GATGCAGCTCCGTGGACC
nad4 intron 1	GTAGAGGATCGACCCGTTCAGTAG	TGGAGTTCGGCCTGTAGAGC
nad4 intron 2	CTCTTATTTGTCAGCCGTGAGG	AGATTTCTCCCGGAGTACGC
nad4 intron 3	GATGGACGTTGCACATGAC	ACCGGATATCAAAACCATAAGG
nad5 intron 1	CCCTTTTCTTGATCCAGCCT	AGTCAGAAGTGAATTACGAGTCGG
nad5 intron 2	CCGAATGGAGGGAGACGTT	TGGAAAGGCTCGGCTTCA
nad5 intron 4	GCCTTCTTTCGTCCGTCCAC	CGGAAAGGCTGCTATACCATACC
nad7 intron 1	GCTCCTATGCCGCTAGCTATG	GACAGCAGGCGGGTTACAC
nad7 intron 2	GATCGCGGAGTCACTGAATG	GTTCCACCGAATGCTCCTATC
nad7 intron 3	TGATGGAACATGGTAAGCCTATCT	CGCAACAGCAGGAAACTATGAC
rps3 intron	CAGCGCGGGTAGCCTACTTAG	CGTTCGGTTCGGATAAGTCAAG
cox2 intron	GTTTAGAGGCCTTATAGTAGCA	TCACGTCAGTACCTCTCAGAA
ccmFc intron	TGCTCAGTTGACTCCTTAACC	TGGTCAAAGACTTGGTTGG
rpl2 intron	AGAGAGGCCAGGCAATGAC	CTACAAGAAGGAAAGCTTAGCGAG

Supplementary Table S2. Primers used for quantitative RT-PCR analysis.

Gene or intron	Spliced transcript		Unspliced transcript	
	**Primer 1**	**Primer 2**	**Primer 1**	**Primer 2**
rpl2 intron	CCGAAGACGGATCAAGGTAA	CGCAATTCATCACCATTTTG	TTAGGAAGAGCCGTACGAGG	CGCAATTCATCACCATTTTG
rps3 intron	AGCCGAAGGTGAGTCTCGTA	CCGATTTCGGTAAGACTTGG	AGCCGAAGGTGAGTCTCGTA	TCTACGGCGGGGTCACTAT
cox2 intron	TGGGGGATTAATTGATTGGA	TGATGCTGTACCTGGTCGTT	TGGGGGATTAATTGATTGGA	AGCAGTACGAGCTGAAAGGC
ccmFc intron	GTGGGTCCATGTAAATGATCG	CACATGGAGGAGTGTGCATC	CCCGGATCGAATCAGAGTT	CACATGGAGGAGTGTGCATC
nad1 intron 1	GACCAATAGATACTTCATAAGAGACCA	TTGCCATATCTTCGCTAGGTG	GACCAATAGATACTTCATAAGAGACCA	CGTGCTCGTACGGTTCATAG
nad1 intron 2	ATTCAGCTTCCGCTTCTGG	TCTGCAGCTCAAATGGTCTC	GGTTGGGTTAGGGGAACATC	TCTGCAGCTCAAATGGTCTC
nad1 intron 3	AAAAGAGCAGACCCCATTGA	TCCGTTTGATCTCCCAGAAG	AAAAGAGCAGACCCCATTGA	GGGAGCTGTATGAGCGGTAA
nad1 intron 4	AGCCCGGGATCTTCTTGA	TCTTCAATGGGGTCTGCTC	AGCCCGGGATCTTCTTGA	ACGGAGCTGCATCCCTACT
nad2 intron 1	GCGAGCAGAAGCAAGGTTAT	GGATCCTCCCACACATGTTC	GCGAGCAGAAGCAAGGTTAT	CCCATTCCTAACCAGTGGAG
nad2 intron 2	AAAGGAACTGCAGTGATCTTGA	AATATTTGATCTTAGGTGCATTTTC	TGTGGTGGTTGGGCCTAC	AAAGGAACTGCAGTGATCTTGA
nad2 intron 3	GCGCAATAGAAAGGAATGCT	CTATGGGTCTACTGGAGCTACCC	GCGCAATAGAAAGGAATGCT	GGCGAATTTCAAACTTGTGG
nad2 intron 4	CAAAGGAGAGGGGTATAGCAA	TATTTGTTCTTCGCCGCTTT	CTTATTCGTGGCAACCTTCC	TATTTGTTCTTCGCCGCTTT
nad4 intron 1	ATTCTATGTTTTTCCCGAAAGC	GAAAAACTGATATGCTGCCTTG	CCGTATGATGCGGAAGTCTC	GAAAAACTGATATGCTGCCTTG
nad4 intron 2	AATACCCATGTTTCCCGAAG	TGCTACCTCCAATTCCCTGT	GCGGAACGACCAGAAAAATA	TGCTACCTCCAATTCCCTGT
nad4 intron 3	TTCCTCCATAAATTCTCCGATT	TGAAATTTGCCATGTTGCAC	TCTAGCTTGGTTCGGAGAGC	TGAAATTTGCCATGTTGCAC
nad5 intron 1	TGGACCAAGCTACTTATGGATG	CCATGGATCTCATCGGAAAT	TGGACCAAGCTACTTATGGATG	TTCGCAAATAGGTCCGACT
nad5 intron 2	AACTCGGATTCGGCAAGAA	CTGGCTCTCGGGAGTCTCTT	GTACGATCGTGTCGGGTGA	CTGGCTCTCGGGAGTCTCTT
nad5 intron 3	AACTCGGATTCGGCAAGAA	CTGGCTCTCGGGAGTCTCTT	AACTCGGATTCGGCAAGAA	GCCGTGTAATAGGCGACCA
nad5 intron 4	AACATTGCAAAGGCATAATGA	GTTCCTGCGTTTCGGATATG	AACATTGCAAAGGCATAATGA	CCTGTAAACCCCCATGATGT
nad7 intron 1	ACCTCAACATCCTGCTGCTC	AAGGTAAAGCTTGAAGATAAGTTTTGT	ACGGTTTTTAGGGGGATCTG	AAGGTAAAGCTTGAAGATAAGTTTTGT
nad7 intron 2	GAGGGACTGAGAAATTAATAGAGTACA	TGGTACCTCGCAATTCAAAA	AGTGGGAGAGCCGTGTTATG	TGGTACCTCGCAATTCAAAA
nad7 intron 3	ACTGTCACTGCACAGCAAGC	CATTGCACAATGATCCGAAG	TAAAGTGAAGTGGTGGGCCT	CATTGCACAATGATCCGAAG
nad7 intron 4	GATCAAAGCCGATGATCGTAA	AGGTGCTTCAACTGCGGTAT	CGGCCAAATGACTACAGGAT	AGGTGCTTCAACTGCGGTAT
cox1	GTAGCTGCGGTGAAGTAGGC	CTGCCTGGATTCGGTATCAT		
rps12	AGCCAAAGTACGGTTGAGCA	TTTGGGTTTTTCTGCACCAT		
ACT (At1g49240, At3g18780)	GGTAACATTGTGCTCAGTGGTGG	AACGACCTTAATCTTCATGCTGC		
18S (At3g41768)	AAACGGCTACCACATCCAAG	ACTCGAAAGAGCCCGGTATT		

Supplementary Table S3. Primers used for determining excised intron configurations.

Intron	RT Primer	PCR Primer 1	PCR Primer 2
rpl2 intron	TTATACCCGCATCTGATCG	GGGGGCTTCAAGTTCTTAGG	GCTCTTTCTAGGTGGGTCGC
rps3 intron	AGCGTTAGAAGAAGTCGTGTC	GGTGACTATCCAGCACGGTTC	AAAAGACTTACCCATTCGGTGAC
cox2 intron	CACTGTAATAGCTTGCTTCTCG	AGCAGTACGAGCTGAAAGGC	CAGAGTGGGGCTCAACAGTC
ccmFc intron	GTTAAGGAGTCAACTGAGCATCT	GGCCCGAGCTGTATGAGG	CCCGGATCGAATCAGAGTT
nad1 intron 2	AACTCAAAATGAGCCTTGC	AGCCACATTGCAGGGAAACT	GGTTGGGTTAGGGGAACATC
nad1 intron 3	CAATAAGACTCGCGCTAGG	GGGAGCTGTATGAGCGGTAA	CCCACCCCTAGTTTCAGTCAG
nad1 intron 4	TGCTTACACACAAGGCTACC	CCGTATGACGCGAGAGTGTC	GAAGCCCAACGAATGTCAGA
nad2 intron 1	CCGCATTAGGTTAGGGTAG	TGATCGGTCTGCTAGTTCACG	GGCTGGGCGATAATAGTTCCT
nad2 intron 2	ATACCCGATCCGATAGTTTACA	CTTTGTGCGAGCCGTATG	AGATATTAATTGATATCGGTAGTTGTCC
nad2 intron 3	AATCTGAGCGGTACCACC	GGCGAATTTCAAACTTGTGG	CCACCCACCCTACCCTACC
nad2 intron 4	AACTGCCTTCGGTCCTC	CAAGTTGGTGAGCCGTATGA	CTTATTCGTGGCAACCTTCC
nad4 intron 1	CTTACACACAAGACTACCCCTC	CCTGAGAAGGGAGTGGCTACC	TTTACGACGACGGACGACC
nad4 intron 2	CTTTTGTTGCGACATGCT	ATGATAGGTGGTAACTATCTTGTACGG	GCTAGTAGGTTGATGGGTCGC
nad4 intron 3	CCAAACCAACTGAGGGAAG	TCTAGCTTGGTTCGGAGAGC	CCATCGCAAGCACCTACA
nad5 intron 1	CAGATCTATCTACTCGGCAATTC	ACGGTTCGGAGAGCACTG	GGCAATTCATCCGGTGAC
nad5 intron 2	TTTCTGCTCACTCGTAGGG	AACCTTAACCGCGCAAGC	TCGTGTCGGGTGAGCAA
nad5 intron 3	GCTATCTTTCCCCTCTTTATTCTC	GCAGAGTTAGTGAGCCGTGTAA	GTGAGTCCCATGCGTCAGT
nad5 intron 4	CTTTCCTCGGGTTCGTAG	CCTGTAAACCCCCATGATGT	CCACGGGTCATAACGCAC
nad7 intron 1	GTAGTAAGGTAGGGCGGTTC	ACGGTTTTTAGGGGGATCTG	TGACTTCGCCCCTTAGCC
nad7 intron 2	ACATGCTGTTGTTGTCGTC	AGTGGGAGAGCCGTGTTATG	CGAAAGAAACGGAGGACTTCA
nad7 intron 3	AGTAGAGTCAGGCGGAACC	TGAAGGGAAACTCTCACGTACA	GGAACCATTGTTGCCTGATG
nad7 intron 4	TGCTTTACTCCTAACCCACA	CGTTAGGTTTGGAGAGAGATGG	ACGGAGCTTAACCTGAGTCTTG

Supplementary Table S4. Sequences of cloned cRT-PCR products shown in Figure 1C.

cRT-PCR product			No. of clones +T	-T
**a**	5' end	**GGCTTGGATTACTTAGTGTTgcgcgcctaggagggcagcg**		
		AGTGTTgcgcgcctaggagggcagcg	2/12	
		gcgcgcctaggagggcagcg	9/12	13/13
		cgcgcctaggagggcagcg	1/12	
	3' end	**aggttcaaagaaagggtaggccgtcggtttgaatccaata**		
		aggttcaaagaaagggtaggc	7/12	4/13
		aggttcaaagaaagggtagg	2/12	3/13
		aggttcaaagaaagggtag		1/13
		aggttcaaagaaagggta	1/12	2/13
		aggttcaaagaaagggt		1/13
		aggttcaaagaaaggg		1/13
		aggttcaaagaaagg	2/12	1/13
**u1**	5' end	**tgagccgaatcactatcattatattatatattcattgttg**		
		tcattatattatatattcattgttg	4/4	3/3
	3' end	**aggttcaaagaaagggtaggccgtcggtttgaatccaata**		
		aggttcaaagaaagggtaggc	3/4	1/3
		aggttcaaagaaagggtagg		
		aggttcaaagaaagggt	1/4	
		aggttcaaagaaagg		2/3

cRT-PCR products are designated a and u1, as in Figure 1. Sequences highlighted in grey are Arabidopsis mtDNA sequences to which we mapped cRT-PCR product ligation sites. Transcript 5' and 3' ends identified from sequencing across ligation sites are displayed underneath, together with the number of clones showing these ends.

For product a, the row labelled 5' end shows the mtDNA sequence at the junction of *nad2* exon 1 (uppercase letters) and intron 1 (lowercase letters). For product u1, the row labelled 5' end corresponds to the mtDNA sequence to which *nad2* mRNA 5' ends have been mapped in previous studies (underlined nucleotides) (43, 58). Rows labelled 3' end display the mtDNA sequence to which *nad2* mRNA 3' ends (underlined nucleotides) have been mapped by Forner et al., 2007 (43).

Product a was analysed for TAP-treated (+T) versus untreated (-T) RNA extracted from *odb1-1* seedlings. Product u1 was analysed for TAP-treated (+T) versus untreated (-T) RNA extracted from wild-type seedlings.

Supplementary Table S5. Analysis of excised, reverse-transcribed and cloned mitochondrial introns.

Intron		No. of clones<br>+L	-L
rpl2	⌈**GGUCA*A***<br>**UGCACAGGGGCUUAGUGUGACCCG**		
replicate 1	TGCACAGGGGCTTTGTGTGACCCG	5/9	
	TGCACAGGGGCTTAGGTCAGTGTGACCCG	3/9	
	TGCACAGGGGCTTAGGTCAACA_6GTGTGACCCG	1/9	
replicate 2	TGCACAGGGGCTTTGTGTGACCCG	8/34	
	TGCACAGGGGCTTAGGTCAGTGTGACCCG	21/34	
	TGCACAGGGGCTTAGGTCAACA_6GTGTGACCCG	5/34	
rps3	⌈**CUCUAU*U***<br>**GGGUCACUAUUGAGGGCGACCGCG**		
replicate 1	GGGTCACTATTGTGGGCGACCGCG	7/24	5/16
	GGGTCACTATTTTGGGCGACCGCG	6/24	1/16
	GGGTCACTATTG GGGCGACCGCG	5/24	1/16
	GGGTCACTATT GGGCGACCGCG	4/24	8/16
	GGGTCACTATATTGGGCGACCGCG	1/24	
	GGGTCACTATTT GGGCGACCGCG		1/16
replicate 2	GGGTCACTATTG GGGCGACCGCG		4/13
	GGGTCACTATT GGGCGACCGCG		9/13
replicate 3	GGGTCACTATTGTGGGCGACCGCG	4/11	10/12
	GGGTCACTATTGAGGGCGACCGCG	1/11	
	GGGTCACTATTG GGGCGACCGCG	5/11	1/12
	GGGTCACTATT GGGCGACCGCG	1/11	1/12
ccmFc	⌈**GGUCAACU**<br>**AGUAAUG*GUGCG*GCUUA*GUGCG*ACCCGGC**		
replicate 1	AGTAATG* GTGCGACCCGGC	15/15	7/7
replicate 2	AGTAATGGTGCGGCTTTGTGCGACCCGGC	2/13	2/16
	AGTAATG* GTGCGACCCGGC	11/13	14/16
replicate 3	AGTAATGGTGCGGCTT GTGCGACCCGGC	2/27	
	AGTAATGGT GTGCGACCCGGC	1	
	AGTAATG* GTGCGACCCGGC	24/27	30/30
	*products potentially deriving from RT-PCR artifacts or incorrect splicing		
cox2	⌈**CCCCUA**<br>**CCAAAGAAAGCUGAGCGCCCGGAA**		
replicate 1	CCAAAGAAAGCTGTGCGCCCGGAA	24/24	16/16

Supplementary Table S5 – continued.

Intron		No. of clones +L	-L
nad1i2	**GGGGACACCCCGGUAUGUGCGCCUUG**		
replicate 1 Col-0	GGGGACACCCCGGTATGTGCGCCTTG	14/25	
	GGGGACACCCCGGTATA$_{10-12}$GTGCGCCTTG	6/25	
	GGGGACACCCCGGTATA$_{7}$CGTGCGCCTTG	2/25	
	GGGGACACCCCGGTATAATAAGTGCGCCTTG	3/25	
replicate 2 Col-0	GGGGACACCCCGGTATGTGCGCCTTG	6/15	
	GGGGACACCCCGGTATA$_{3-7}$GTGCGCCTTG	2/15	
	GGGGACACCCCGGTATA$_{5}$CGTGCGCCTTG	1/15	
	GGGGACACCC GTGCGCCTTG	4/15	
	GGGGACAC GTGCGCCTTG	1/15	
	GGGAAA GTGCGCCTTG	1/15	
replicate 3 Col-0	GGGGACACCCCGGTATGTGCGCCTTG	17/32	14/14
	GGGGACACCCCGGTATA$_{1-6}$GTGCGCCTTG	3/32	
	GGGGACACCCCGGTATA$_{5}$CGTGCGCCTTG	1/32	
	GGGGACACCCCGGTATA$_{9}$CA$_{5}$GTGCGCCTTG	1/32	
	GGGGACACCCC GTGCGCCTTG	6/32	
	GGGGACACCC GTGCGCCTTG	4/32	
replicate 4 Col-0	GGGGACACCCCGGTATGTGCGCCTTG	17/37	7/17
	GGGGACACCCAGGTATGTGCGCCTTG		1/17
	GGGGACACCCCGGTATA$_{3-9}$GTGCGCCTTG	4/37	
	GGGGACACCCCGGTATA$_{3}$CGTGCGCCTTG	1/37	
	GGGGACACCCCGGTATA$_{4}$CA$_{4}$GTGCGCCTTG	1/37	
	GGGGACACCCC GTGCGCCTTG	6/37	
	GGGGACACCTC GTGCGCCTTG	1/37	
	GGGGACACCC GTGCGCCTTG	7/37	
	GGG GTGCGCCTTG		1/17
	GG GTGCGCCTTG		8/17
replicate 1 *odb1-1*	GGGGACACCCCCGGTATGTGCGCCTTG	12/32	35/35
	GGGGACACCCCCGGTGTGTGCGCCTTG	1/32	
	GGGGACACCCCCGGTATA$_{3-17}$GTGCGCCTTG	7/32	
	GGGGACACCCCCGGTATA$_{6}$CA$_{5}$GTGCGCCTTG	1/32	
	GGGGACACCCC GTGCGCCTTG	5/32	
	GGGGACACCC GTGCGCCTTG	6/32	

Supplementary Table S5 – continued.

Intron		No. of clones<br>+L	 -L
nad1i3	⌠**CUCUCG**<br>**CUUGUUGUACCUUAGCGCGGGAUG**		
replicate 1	CTTGTTGTACCTTTGCGCGGGATG	18/23	11/16
	CTTGTTGTACCCTTGCGCGGGATG	1/23	
	CTTGTTGTACCTT GCGCGGGATG	4/23	2/16
	CTTGTTGTACCCTCGCGCGGGATG		1/16
	CTTGTTGTACCTTACTCTCGGCGCGGGATG		2/16
replicate 2	CTTGTTGTACCTTTGCGCGGGATG		10/17
	CTTGTTGTACCTT GCGCGGGATG		1/17
	CTTGTTGTACCCTCGCGCGGGATG		1/17
	CTTGTTGTACCG GCGCGGGATG		1/17
	CTTGTTGTACCCTCGGCGCGGGATG	1/15	
	CTTGTTGTACCTTACTCTCGGCGCGGGATG	15/16	4/17
nad1i4	⌠**CUCACC**<br>**GAGCUGCAUCCUUAGUGCGGAACU**		
replicate 1	GAGCTGCATCCTTTGTGCGGAACT	10/12	13/19
	GAGCTGCATCCTT GTGCGGAACT		1/19
	GAGCGGTCCAC GTGCGGAACT		1/19
	AGGCCCGACGA GTGCGGAACT		1/19
	CCCTACTCACC GTGCGGAACT	2/12	1/19
	GTTTCTTTGAG GTGCGGAACT		1/19
	GAGCTGCATCCTTAAGTGCGGAACT		1/19
	Several additional products were detected in the –L sample in which the joined 5' and 3' ends were incorrect by a few nucleotides at 5' and ~30 nt at 3' end; these might derive from mis-splicing events.		
replicate 2	GAGCTGCATCCTTTGTGCGGAACT	7/9	4/6
	GAGCTGCATCCTT GTGCGGAACT	2/9	
	GAGCTGCACCCTTCGTGCGGAACT		1/6
	GAGCTGCATCCTTACTCACC*TG*GTGCGGAACT		1/6

Supplementary Table S5 – continued.

Intron		No. of clones +L	-L
nad2i1	**TCTGGCCGGGCUUUCCUGAGGUAUGCGCGCCUAG**		
replicate 1 Col-0	TCTGGCCGGGCTTTCCTGAGGTATGCGCGCCTAG	14/20	
	TCTGGCCGGGCTTTCCTGAGGTATA$_5$CGCGCGCCTAG	1/20	
	TCTGGCCGGGCTTTCCTGAGGTATA$_5$CACGCGCGCCTAG	1/20	
	TCTGGCCGGGCT GCGCGCCTAG		11/14
	TCTGGCCGG GCGCGCCTAG	3/20	3/14
	TCTG GCGCGCCTAG	1/20	
replicate 2 Col-0	TCTGGCCGGGCTTTCCTGAGGTATGCGCGCCTAG	17/31	
	TCTGGCCGGGCTTTCCTGAGGTAT CGCGCCTAG	3/31	
	TCTGGCCGGGCTTTCCTGAGGT GCGCGCCTAG	1/31	
	TCTGGCCGGGCTTTCCT GCGCGCCTAG	3/31	
	TCTGGCCGGGCTTTC GCGCGCCTAG	1/31	
	TCTGGCCGGGCTTT GCGCGCCTAG	1/31	
	TCTGGCCGGGCT GCGCGCCTAG	1/31	
	TCTGGCCGGG GCGCGCCTAG	2/31	1/26
	TCTGGCCGG GCGCGCCTAG	1/31	23/26
	TCTGGCCGGA CGCGCCTAG		1/26
	TCTGGCCG GCGCGCCTAG		1/26
	TCTGGCC GCGCGCCTAG	1/31	
replicate 3 Col-0	TCTGGCCGGGCTTTCCTGAGGTATGCGCGCCTAG	8/19	
	TCTGGCCGGGCTTTCCTGAGGTATA$_{1-15}$GCGCGCCTAG	10/19	
	TCTGGCCGG GCGCGCCTAG	1/19	6/7
	TCTGGCC GCGCGCCTAG		1/7
replicate 1 *odb1-1*	TCTGGCCGGGCTTTCCTGAGGTATGCGCGCCTAG	12/23	
	TCTGGCCGGGCTTTCCTGAGGTATTGCGCGCCTAG	2/23	
	TCTGGCCGGGCTTTCCTG GCGCGCCTAG		1/24
	TCTGGCCGGGCTTT GCGCGCCTAG	1/23	
	TCTGGCCGGGCTT GCGCGCCTAG		1/24
	TCTGGCCGGG GCGCGCCTAG	2/23	3/24
	TCTGGCCGG GCGCGCCTAG	2/23	13/24
	TCTGGCC GCGCGCCTAG	4/23	
	TCTGG GCGCGCCTAG		6/24
replicate 2 *odb1-1*	TCTGGCCGGGCTTTCCTGAGGTATGCGCGCCTAG	6/19	2/4
	TCTGGCCGGGCTTTCCTGAGGTATA$_{2-4}$GCGCGCCTAG	3/19	
	TCTGGCCGGGCTTTCCTGAGGTATA$_3$TA$_5$GCGCGCCTAG	1/19	
	TCTGGCCGGGCTTTCCTGAGGTA GCGCGCCTAG	1/19	
	TCTGGCCGGGCTTTCCTGAGGTAA$_{3-8}$GCGCGCCTAG	5/19	
	TCTGGCCGGGCTTTCCTGAGGTAA$_3$CGCGCGCCTAG	1/19	
	TCTGGCCGGGCTTTCCTGAGGTAA$_7$CA$_4$GCGCGCCTAG	1/19	
	TCTGGCCGGGCTTT GCGCGCCTAG	1/19	
	TCTGGCCGGG GCGCGCCTAG		1/4
	TCT GCGCGCCTAG		1/4

Supplementary Table S5 – continued.

Intron		No. of clones	
		+L	-L
nad2i2	CCCUAU<br>**GUUGGGCCUACCCAGUACGACCGG**		
replicate 1	GTTGGGCCTACCCTGTACGACCGG	13/15	13/13
	GTTGGGCCTACCC GTACGACCGG	1/15	
	GTTGGGCCTACCCCGTACGACCGG	1/15	
nad2i3	CUCUAU<br>**CAUAUUCCCUUUGAGGGCGGCUGU**		
replicate 1	CATATTCCCTTTGTGGGCGGCTGT	7/22	12/23
	CATATTCCCTTTGAGGGCGGCTGT	1/22	
	CATATTCCCTTT GGGCGGCTGT	1/22	1/23
	CATATTCCCTTTT GGGCGGCTGT	6/22	3/23
	CATATTCCCTTTTTGGGCGGCTGT	1/22	2/23
	CATATTCCCTTTTTTGGGCGGCTGT		1/23
	CATATTCCCTTTATGGGCGGCTGT	1/22	
	CATATTCCCTTTG GGGCGGCTGT	4/22	2/23
	CATATTCCCTTTTGGGGCGGCTGT		1/23
	CATATTCCCTTTTTGGGGCGGCTGT		1/23
	CATATTCCCTTTGCGGGCGGCTGT	1/22	
nad2i4	CUCUA<br>**UGGACCCUUUUUCAGGGCGGCCGG**		
replicate 1	TGGACCCTTTTTCTGGGCGGCCGG	21/23	20/21
	TGGACCCTTTT CTGGGCGGCCGG	1/23	
	TGGACCCTTTTT TGGGCGGCCGG	1/23	
	TGGACCCTTTTTCCGGGCGGCCGG		1/21
nad4i1	CUCUAC<br>**GGGGCCACCCCUUAGUGCGGAGCG**		
replicate 1	GGGGCCACCCCTTTGTGCGGAGCG	9/13	13/14
	GGGGCCACCCCT TGTGCGGAGCG	2/13	
	GGGGCCACCACTTAGTGCGGAGCG	1/13	1/14
	GGGGCCACCCCTTACTCTACGTGCGGAGCG	1/13	
replicate 2	GGGGCCACCCCTTTGTGCGGAGCG	12/20	13/13
	GGGGCCACCCCTTACTCTACA$_{3-7}$GTGCGGAGCG	7/20	
	GGGGCCACCCCTTACTCTACA$_5$CA$_6$GTGCGGAGCG	1/20	

Supplementary Table S5 – continued.

**Intron**		**No. of clones**<br>**+L**	**-L**
nad4i2	**GGAGAAATCTTTCGCTCTATGGGCGGCGGCCGTT**		
replicate 1	GGAGAAATCTTTCGCTCTATA_{3-14}GGGCGGCGGCCGTT	7/22	
	GGAGAAATCTTTCGCTCTATA_8GA_5GGGCGGCGGCCGTT	1/22	
	GGAGAAATCTTTCGCTCTATA_9TA_7GGGCGGCGGCCGTT	1/22	
	GGAGAAATCTTTCGCTCTATA_8CGGGCGGCGGCCGTT	2/22	
	GGAGAA GGGCGGCGGCCGTT	11/22	
replicate 2	GGAGAAATCTTTCGCTCTA GGGCGGCGGCCGTT	2/12	
	GGAGAAATCTTTCGCT GGGCGGCGGCCGTT	1/12	
	GGAGAAAA_4 GGGCGGCGGCCGTT	4/12	
	GGAGAAA GGGCGGCGGCCGTT	1/12	
	GGAGA GGGCGGCGGCCGTT	3/12	
	GG GGGCGGCGGCCGTT	1/12	
	GTTCGGGGGGT GGGCGGCGGCCGTT		1/11
	GTTCGGGGGGTAA GGGCGGCGGCCGTT		5/11
	GTTCGGGGGGTAATCG GGGCGGCGGCCGTT		5/11
replicate 3	GGAGAAATCTTTCGCTCTATGGGCGGCGGCCGTT	3	
	GGAGAAATCTTTCGCTCTATA_{3-8}GGGCGGCGGCCGTT	1	
	GGAGAAATCTTTA_4T GGGCGGCGGCCGTT	3	
	GGAA_2 GGGCGGCGGCCGTT	6	
	G GGGCGGCGGCCGTT	1	
nad4i3	**CUCUA**<br>**UGCUGAACUAUUGAGGGCGACCG**		
replicate 1	TGCTGAACTATTGTGGGCGACCG	9/14	9/16
	TGCTGAACTATTG GGGCGACCG	4/14	
	TGCTGAACTATT GGGCGACCG	1/14	3/16
	TGCTGAACTATATTGGGCGACCG		2/16
	TGCTGAACTATTTTGGGCGACCG		1/16
	TGCTGAACTATT TGGGCGACCG		1/16

Supplementary Table S5 – continued.

Intron		No. of clones +L	-L
nad5i1	⌠CUCUGU<br>**UAAUUCACUUUUGAGGCGACCGUG**		
replicate 1	TAATTCACTTTTGTGGCGACCGTG	10/14	8/15
	TAATTCACTTCTGTGGCGACCGTG	1/14	3/15
	TAATTCACTTTTTTGGCGACCGTG	1/14	1/15
	TAATTCACTTTTCTGGCGACCGTG		1/15
	TAATTCACTTTTGTTGGCGACCGTG	1/14	
	TAATTCACTTTTTTTGGCGACCGTG	1/14	2/15
nad5i2	⌠GGGACC<br>**AGCAAUGGGGCUUAGUGUGACCCG**		
replicate 1	AGCAATGGGGCTTTGTGTGACCCG	15/15	28/30
	AGCAATGGGGCT TGTGTGACCCG		2/30
nad5i3	⌠CCCCUAU<br>**CGACUGCGUCUUGAGGGCGGCCGG**		
replicate 1	CGACTGCGTCTTGTGGGCGGCCGG	3/22	5/14
	CGACTGCGTCTTGAGGGCGGCCGG		3/14
	CGACTGCGTCTT GGGCGGCCGG	19/22	6/14
replicate 2	CGACTGCGTCTTGTGGGCGGCCGG	7/21	7/21
	CGACTGCGTCTTG GGGCGGCCGG	1/21	2/21
	CGACTGCGTCTT GGGCGGCCGG	13/21	12/21
nad5i4	⌠CCCUAC<br>**CGGGGAUAUCCCGAGUGCGAAGAG**		
replicate 1	CGGGGATATCCCGTGTGCGAAGAG		9/15
	CGGGGATATCCCG GTGCGAAGAG		3/15
	CGGGGATATCCC AGTGCGAAGAG		1/15
	CGGGGATATCCCGAGTGCGAAGAG		1/15
	CGGGGATATCCCCAGTGCGAAGAG		1/15
replicate 2	CGGGGATATCCCGTGTGCGAAGAG		12/12

Supplementary Table S5 – continued.

Intron		No. of clones<br>+L	 -L
nad7i1	**CCGGCCGGCGCCCACCCGAC**GUGCGGCACG		
replicate 1	CCGGCCGGCGCCCACCCGACA$_{7-16}$ GTGCGGCACG	12/14	
	CCGGCCGGCGCCCACCCGACA$_{5}$C GTGCGGCACG	1/14	
	CCGGCCGGCGCCCACCCGACA$_{2-16}$CA$_{2-6}$GTGCGGCACG	1/14	
replicate 2	CCGGCCGGCGCCCACCCGACA$_{7-16}$ GTGCGGCACG	8/15	
	CCGGCCGGCGCCCACCCGACA$_{4}$C GTGCGGCACG	1/15	
	CCGGCCGGCGCCCACCCGACA$_{2-16}$CA$_{3-6}$GTGCGGCACG	6/15	
replicate 3	CCGGCCGGCGCCCACCCGACA$_{7-16}$ GTGCGGCACG	17/35	
	CCGGCCGGCGCCCACCCGACA$_{3}$CACA$_{2}$CA$_{8}$GTGCGGCACG	1/35	
	CCGGCCGGCGCCCACCCGACA$_{7}$CA$_{5}$TA$_{10-11}$GTGCGGCACG	2/35	
	CCGGCCGGCGCCCACCCGACA$_{13}$TA$_{10-11}$ GTGCGGCACG	2/35	
	CCGGCCGGCGCCCACCCGACA$_{9}$GA$_{5}$ GTGCGGCACG	1/35	
	CCGGC GTGCGGCACG	12/35	
	GGCCGAAAG GTGCGGCACG		16/16
nad7i2	**CCCUAU**<br>**CGUUCCGUUUUUGA**GGGCGAUCGC		
replicate 1	CGTTCCGTTTTTGTGGGCGATCGC	7/16	7/14
	CGTTCCGTTTTTT GGGCGATCGC	5/16	2/14
	CGTTCCGTTTTTG GGGCGATCGC	4/16	5/14
nad7i3	**UCCCAAC**<br>**GGUGGGCCUACCCA**GUGCGACAUG		
replicate 1	GGTGGGCCTACCCTGTGCGACATG	12/13	16/16
	GGTGGGCCTACCC GTGCGACATG	1/13	
nad7i4	**CUCUAC**<br>**GGAUCAUCGGUCUA**GUGCGUCGUG		
replicate 1	GGATCATCGGTCTTGTGCGTCGTG	15/15	15/16
	GGATCATCGGTCT GTGCGTCGTG		1/16

Introns are designated as in Figure 4. Rows highlighted in grey show sequences at branch point regions for every analysed mitochondrial intron, with the intron 5' end printed in grey, the predicted branch point ("bulged A") in green, the intron 3' sequence upstream of the predicted branch points in black and the predicted lariat tail in blue. Orange nucleotides mark positions that undergo C-to-U editing. Below each branch point model, sequences obtained from cRT-PCR/RT-PCR experiments are shown. For the majority of introns, several independent experiments were performed. The number of clones identified for every sequence is indicated in columns +L (ligase-treated RNA samples) and –L (untreated samples). Black, grey, green and blue nucleotides in these sequences are as in branch point region models. As previously described (45), sequences of lariat-derived cDNAs show a thymidine instead of adenosine at the branch point. Red letters denote non-encoded nucleotides identified in cRT-PCR and RT-PCR products.
Clones showing the complete intron 3' sequence correspond to introns spliced via a hydrolytic pathway (e.g., *nad1* intron 2) whereas clones lacking the lariat tail nucleotides correspond to introns using the lariat pathway (e.g., *cox2* intron). Sequences including the lariat tail but derived from introns that do not require ligation prior to amplification correspond to introns released via first-step hydrolysis

in a circular form. Thus, both linear and circular forms of excised *nad2* intron 1 were detected. The stretches of non-encoded A and C nucleotides seen in ligase-dependent sequences have been proposed to be added to excised linear introns in order to tag these molecules for the bacterial-type RNA degradation pathway (33).

For several introns, a number of products were obtained with intron 3' sequences that were truncated and shorter than expected for a lariat or hydrolytic pathway; these products could be due to either mis-splicing or RT-PCR artifacts. The corresponding intron molecules are unlikely to have been released as lariats as no mis-reading of the potential alternative branch point nucleotide is seen. All experiments were done on wild-type (Col-0) RNA except experiments for *nad1* intron 2 and *nad2* intron 1, which were additionally performed on RNA extracted from *odb1-1*.
